# The *Clp1* R140H mutation alters tRNA metabolism and mRNA 3′ processing in mouse models of pontocerebellar hypoplasia

**DOI:** 10.1073/pnas.2110730118

**Published:** 2021-09-21

**Authors:** Caitlin E. Monaghan, Scott I. Adamson, Mridu Kapur, Jeffrey H. Chuang, Susan L. Ackerman

**Affiliations:** ^a^Department of Cellular and Molecular Medicine, Division of Biological Sciences, Section of Neurobiology, University of California San Diego, La Jolla, CA 92093;; ^b^HHMI, University of California San Diego, La Jolla, CA 92093;; ^c^The Jackson Laboratory for Genomic Medicine, Farmington, CT 06030;; ^d^Department of Genetics and Genome Sciences, Institute for Systems Genomics, UConn Health, Farmington, CT 06030

**Keywords:** motor neuron degeneration, tRNA splicing, cleavage factor II, deep cerebellar nuclei, alternative polyadenylation

## Abstract

Homozygous mutation of the RNA kinase *CLP1* (cleavage factor polyribonucleotide kinase subunit 1) causes pontocerebellar hypoplasia type 10 (PCH10), a pediatric neurodegenerative disease. CLP1 is associated with the transfer RNA (tRNA) splicing endonuclease complex and the cleavage and polyadenylation machinery, but its function remains unclear. We generated two mouse models of PCH10: one homozygous for the disease-associated *Clp1* mutation, R140H, and one heterozygous for this mutation and a null allele. Both models exhibit loss of lower motor neurons and neurons of the deep cerebellar nuclei. To explore whether *Clp1* mutation impacts tRNA splicing, we profiled the products of intron-containing tRNA genes. While mature tRNAs were expressed at normal levels in mutant mice, numerous other products of intron-containing tRNA genes were dysregulated, with pre-tRNAs, introns, and certain tRNA fragments up-regulated, and other fragments down-regulated. However, the spatiotemporal patterns of dysregulation do not correlate with pathogenicity for most altered tRNA products. To elucidate the effect of *Clp1* mutation on precursor messenger RNA (pre-mRNA) cleavage, we analyzed poly(A) site (PAS) usage and gene expression in *Clp1*^*R140H/−*^ spinal cord. PAS usage was shifted from proximal to distal sites in the mutant mouse, particularly in short and closely spaced genes. Many such genes were also expressed at lower levels in the *Clp1*^*R140H/−*^ mouse, possibly as a result of impaired transcript maturation. These findings are consistent with the hypothesis that select genes are particularly dependent upon CLP1 for proper pre-mRNA cleavage, suggesting that impaired mRNA 3′ processing may contribute to pathogenesis in PCH10.

## Significance

Mutation of *CLP1* causes pontocerebellar hypoplasia type 10 (PCH10), a neurodegenerative disorder associated with intellectual and motor disability. We made two mouse models of PCH10: one homozygous for the mutation found in patients and one heterozygous for this mutation and a null allele. Mutant mice had motor impairments and neurodegeneration in the spinal cord and cerebellum. Mutants also had altered tRNA metabolism; however, it is not clear whether these alterations contribute to pathogenesis. In addition, mutation of *Clp1* resulted in altered poly(A) site selection and gene expression, suggesting that the role of CLP1 in mRNA 3′ end processing could be a promising avenue for future research into the pathogenesis of PCH10.

Homozygosity for the R140H (arginine to histidine) missense mutation in the RNA kinase *CLP1* (cleavage factor polyribonucleotide kinase subunit 1) causes the pediatric disorder pontocerebellar hypoplasia type 10 (PCH10) (1, 2). PCH10 patients are microcephalic, with reduced cortical, and in some cases pontine and/or cerebellar, volume. In addition, patients have severe intellectual disability, epilepsy, and motor dysfunction, including altered reflexes, abnormal muscle tone, and the inability to walk or sit independently (1–3). Like PCH10 patients, *Clp1*^*K127A/K127A*^ mice, in which CLP1 kinase activity is severely impaired, have reduced brain volume and experience motor dysfunction; these phenotypes are due, in part, to progressive loss of cortical neurons and lower motor neurons, respectively (1, 4). In vitro, metazoan CLP1 phosphorylates the 5′-OH of single-stranded RNA, double-stranded RNA, and double-stranded DNA (5). However, both its function in vivo and how disruption of this function results in neuron death remain unclear.

CLP1 is a component of two endonucleolytic complexes: the transfer RNA (tRNA) splicing endonuclease (TSEN) complex and the cleavage and polyadenylation complex (6, 7). The TSEN complex—the core components of which are TSEN2, TSEN34, TSEN54, and TSEN15—initiates tRNA splicing in the minority of pre-tRNAs that contain an intron by cleaving on both sides of this element (6). Ligation of the two exons is then performed by RTCB (RNA 2′,3′-cyclic phosphate and 5′-OH ligase), which joins the 2′,3′-cyclic phosphate of the 5′ exon to the 5′-OH of the 3′ exon (8). CLP1 has been hypothesized to stabilize the TSEN complex and thus facilitate intron excision (4). Consistent with this hypothesis, cleavage activity on an exogenous substrate was impaired in nuclear extracts from *Clp1*^*K127A/K127A*^ (kinase-deficient) mouse and *CLP1*^*R140H/R140H*^ human fibroblasts (1, 4); however, cleavage of a similar pre-tRNA by the core TSEN complex was not improved by the addition of recombinant CLP1 (9). CLP1 has also been hypothesized to phosphorylate the 5′-OH of 3′ exons, which would be predicted to impede tRNA ligation (9), but could instead promote ligation if the phosphorylation occurred in the context of a noncanonical, phosphorylation-dependent pathway analogous to that used in yeast (10). Evidence regarding the contribution of CLP1 to tRNA exon ligation is mixed: *CLP1*-knockdown HeLa cell extracts displayed impaired ligation of free tRNA exons (5), extracts from *CLP1*^*R140H/R140H*^ human fibroblasts had normal ligation activity (1), and knockdown of the *CLP1* homolog *cbc* in *Drosophila* increased levels of mature tRNA generated from an intron-containing tRNA reporter (9). In addition to a potential role in pre-tRNA processing, it has also been suggested that CLP1 phosphorylates the excised intron to enable its exonucleolytic degradation: intron degradation in yeast is dependent upon intron phosphorylation, but whether metazoans employ a similar pathway is unknown (11). Whatever the mechanism, it is apparent that CLP1 influences levels of tRNA gene products, as altered levels of premature and mature tRNAs, free introns, and tRNA fragments (tRFs) were observed in homozygous R140H and K127A cells and tissues (1, 2, 4). However, these effects were not consistent across experiments, and differences in organism, cell type, mutation, and experimental approach hinder interpretation of these results and thus ascertainment of the function of CLP1 within the TSEN complex.

In addition to being a member of the TSEN complex, CLP1 is part of the cleavage/polyadenylation machinery, which cleaves each precursor messenger RNA (pre-mRNA) at a polyadenylation site (PAS) and synthesizes a poly(A) tail on the 3′ end of the resultant upstream RNA (12). CLP1 and its binding partner PCF11 constitute cleavage factor II (CFII), which was essential for pre-mRNA cleavage in an in vitro assay, although whether CLP1 itself was necessary was not tested (13). Whether CFII is universally required for pre-mRNA cleavage cannot be readily ascertained because both its constituent proteins are absolutely required for cell survival; however, many studies have demonstrated that CFII influences where cleavage occurs. Knockdown of *CLP1* in human cell lines and mouse thymic cells increased the relative use of distal PASs, while in contrast, *Clp1* knockdown in mouse myoblast cells caused a slight bias toward use of proximal PASs (14–17). Knockdown of *PCF11* in human and mouse cells and knockout in zebrafish also influenced PAS selection, with a shift toward use of distal sites (14–16, 18, 19). Furthermore, knockdown of *PCF11* in human and mouse cells also caused down-regulation of both closely spaced and short genes, suggesting that such genes might be especially dependent upon PCF11 for proper 3′ end maturation (18, 19). In conjunction with studies showing that CLP1 and PCF11 levels vary between tissues (17, 18), these findings suggest that CFII may not be universally required for pre-mRNA cleavage, but rather contribute to cleavage in a transcript- and/or cell-type specific manner. Whether the *Clp1* R140H mutation influences mRNA 3′ processing and gene expression is unknown.

To investigate the effect of the R140H mutation in *Clp1* on tRNA processing and mRNA cleavage in vivo, we generated and characterized two mouse models of PCH10. We show that mice homozygous for the *Clp1* R140H mutation and mice heterozygous for this mutation and a null *Clp1* mutation had motor impairments and progressive loss of neurons in the spinal cord and in the deep cerebellar nuclei (DCN). In both models, levels of mature tRNAs were normal. However, intron-containing pre-tRNAs were frequently up-regulated in neural tissues, and levels of certain tRFs and introns were altered. Changes in the abundance of pre-tRNAs and tRFs were also observed in an intronless tRNA family, which calls into question the prevailing presumption that the impact of *Clp1* mutation upon tRNA metabolism is directly due to perturbation of tRNA splicing. Mutation of *Clp1* also caused a shift toward use of distal PASs, which is consistent with the hypothesis that CLP1 improves the efficiency of mRNA cleavage. Furthermore, mutation of *Clp1* altered gene expression in a manner correlating with gene biotype, density, and length, which suggests that CLP1 may be especially important for the 3′ end processing of short, closely spaced genes. Although the neural phenotypes associated with *CLP1* mutation have been previously attributed to impairment of tRNA splicing, our results raise the possibility that defects in mRNA cleavage could contribute to pathogenesis.

## Results

### Progressive Motor Impairment in *Clp1* Mutant Mice.

To investigate the effects of the *Clp1* R140H mutation in vivo, we introduced the mutation into C57BL/6J mice. *Clp1*^*R140H/R140H*^ mice were present at expected numbers at weaning (27% of mice from heterozygous matings, χ^2^ *P* = 0.83, *n* = 178). The weights of *Clp1*^*R140H/R140H*^ mice were normal at postnatal day 14 (P14) (*SI Appendix*, Fig. S1). At 1 mo of age, mutant females, but not males, weighed less than wild-type mice, and both sexes weighed 21 to 30% less than wild-type controls at 5 and 9 mo. In addition, *Clp1*^*R140H/R140H*^ mice developed a halting gait and mild tremor by 4 mo of age.

Reasoning that the effects of the R140H mutation might be exacerbated by lower gene dosage, we generated compound heterozygotes with an R140H and a null *Clp1* allele. Mice with the null allele had a 16-nt deletion in the second of three exons, creating a frameshift after codon 139 (of 425), which was shortly followed by a termination codon. Consistent with a previous report of early lethality in *Clp1*-null embryos (4), *Clp1*^*−/−*^ embryos were not recovered from heterozygous matings at embryonic day (E) 7.5 (0 of 43 embryos). *Clp1*^*R140H*^^*/*^*^−^* (compound heterozygote) pups from *Clp1*^*+/−*^ by *Clp1*^*R140H/+*^ matings were present at the expected frequency at P0 (28% of pups, χ^2^ *P* = 0.32, *n* = 194). However, at 1 to 2 wk of age, fewer *Clp1*^*R140H/−*^ pups were observed (16% of pups, χ^2^ *P* = 0.0004, *n* = 380), which suggests that this combination of mutations confers substantial risk of mortality during the first 2 wk after birth. Although surviving *Clp1*^*R140H/−*^ mice were normal in appearance and weight at P14, by 1 mo of age, *Clp1*^*R140H/−*^ mice had developed a tremor and altered gait, and weighed less than controls (*SI Appendix*, Fig. S1). By 5 mo, these mice had severe kyphosis, curled forepaws, and rigid, splayed hindlimbs, and were half the weight of wild-type mice; at this time, the severity of motor dysfunction and poor body condition necessitated euthanasia.

**Fig. 1.**
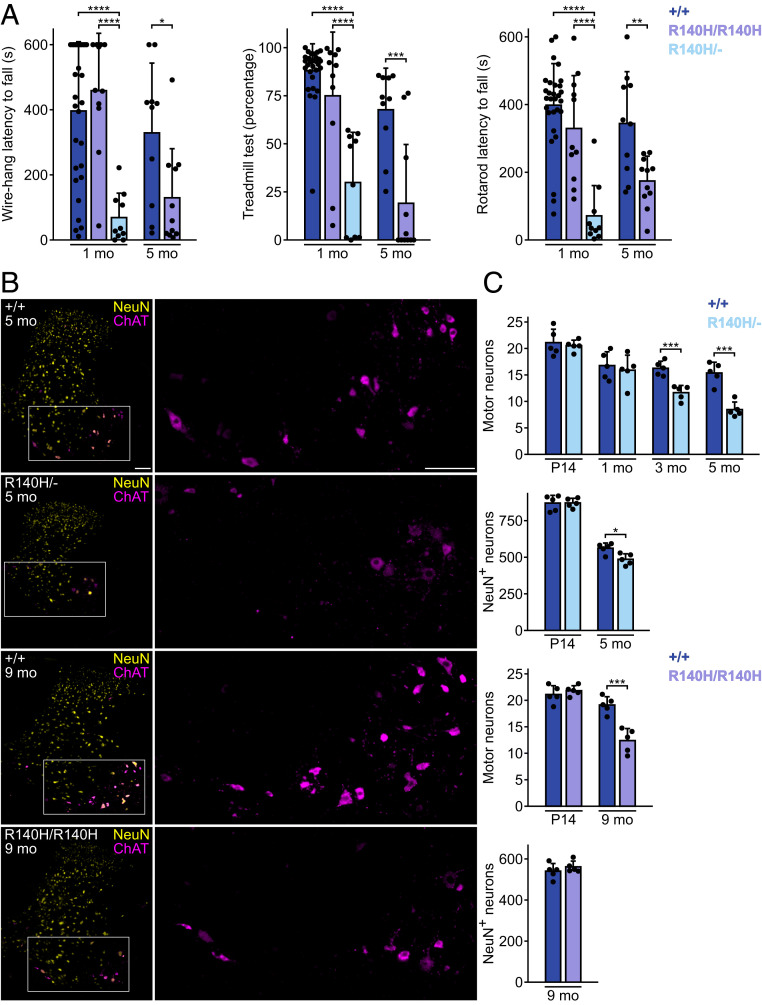
*Clp1* mutant mice have motor function impairment and spinal cord neuron loss. (*A*) Motor tests. (*Left*) Wire-hang average latency to fall across three trials. (*Center*) Treadmill walking time expressed as a percentage of time facing forward. (*Right*) Rotarod average latency to fall across three trials. One-way ANOVA with Tukey’s multiple comparisons tests (1-mo-old mice) and *t* tests with Welch’s correction (5-mo-old mice). (*B*) Immunofluorescence for ChAT and NeuN in C6 cervical spinal cord. High-magnification images (*Right*) of ChAT in boxed region. (Scale bars, 100 µm.) (*C*) Average number of cells per hemisection of C6 spinal cord. *t* tests with Welch’s correction. Mean + SD. *P ≤ 0.05; **P ≤ 0.01; ***P ≤ 0.001; ****P ≤ 0.0001.

To analyze the effect of *Clp1* mutations on motor function, we performed rolling wire-hang, treadmill, and accelerating rotarod tests on 1-mo-old wild-type, *Clp1*^*R140H/R140H*^, and *Clp1*^*R140H/−*^ mice (Fig. 1*A*). In the rolling wire-hang test, which probes grip strength, the duration a mouse hung from a loop of wire was recorded. Most wild-type and *Clp1*^*R140H/R140H*^ mice suspended themselves from the wire for the full duration (10 min) in at least one of three trials; in contrast, no *Clp1*^*R140H/−*^ mouse reached the halfway point during any trial. In the treadmill task, we measured the percentage of time that a mouse kept ahead of the bumper at the rear of the apparatus while the treadmill ran at a constant speed. Wild-type and *Clp1*^*R140H/R140H*^ mice both performed well, with few mice walking less than three-quarters of the time. Conversely, *Clp1*^*R140H/−*^ mice were dramatically impaired, with the average mouse walking for less than one-third of the time. In the accelerating rotarod test, the latencies to fall of wild-type and *Clp1*^*R140H/R140H*^ mice were not significantly different. *Clp1*^*R140H/−*^ mice performed poorly on this task, with a latency to fall of 20% of that of wild-type mice.

We next analyzed the performance of 5-mo-old wild-type and *Clp1*^*R140H/R140H*^ mice. *Clp1*^*R140H/−*^ mice were excluded because they were uniformly incapable of performing the tests at this age. *Clp1*^*R140H/R140H*^ mice performed poorly in all three tests, with wire-hang durations, treadmill walking percentages, and rotarod durations of 39%, 28%, and 51% of wild-type values, respectively. Collectively, these data demonstrate progressive motor dysfunction in both genotypes, with earlier onset and increased severity in *Clp1*^*R140H/−*^ mice.

### Degeneration of Spinal Cord Neurons in *Clp1* Mutant Mice.

To explore whether mice with the *Clp1*^*R140H*^ mutation have degeneration of lower motor neurons as observed in *Clp1*^*K127A/K127A*^ mice and suspected in PCH10 patients (2, 4), we counted cervical spinal motor neurons, identified on the basis of their location and choline acetyltransferase (ChAT) positivity (Fig. 1 *B* and *C*). *Clp1*^*R140H/−*^ mice had normal numbers of motor neurons at P14 and 1 mo of age, but 28% and 45% fewer than wild-type mice at 3 and 5 mo, respectively. Noting that the spinal cord appeared smaller in *Clp1*^*R140H/−*^ mice, we also counted cells positive for NeuN, which is expressed by a majority of neurons. While *Clp1*^*R140H/−*^ mice had normal numbers of these cells at P14, 5-mo-old *Clp1*^*R140H/−*^ mice had 14% fewer NeuN^+^ cells than wild-type mice, which suggests that additional types of cervical spinal cord neurons also degenerate in these mice.

We determined if the spinal cord of *Clp1*^*R140H/R140H*^ mice also had fewer motor neurons and NeuN^*+*^ neurons. Normal numbers of motor neurons were observed in these mice at P14, but 35% fewer were present in mutant than in wild-type mice at 9 mo. However, in contrast to *Clp1*^*R140H/−*^ mice, numbers of NeuN^*+*^ neurons were normal in aged *Clp1*^*R140H/R140H*^ mice.

### Death of Neurons in the DCN in *Clp1* Mutant Mice.

**Fig. 2.**
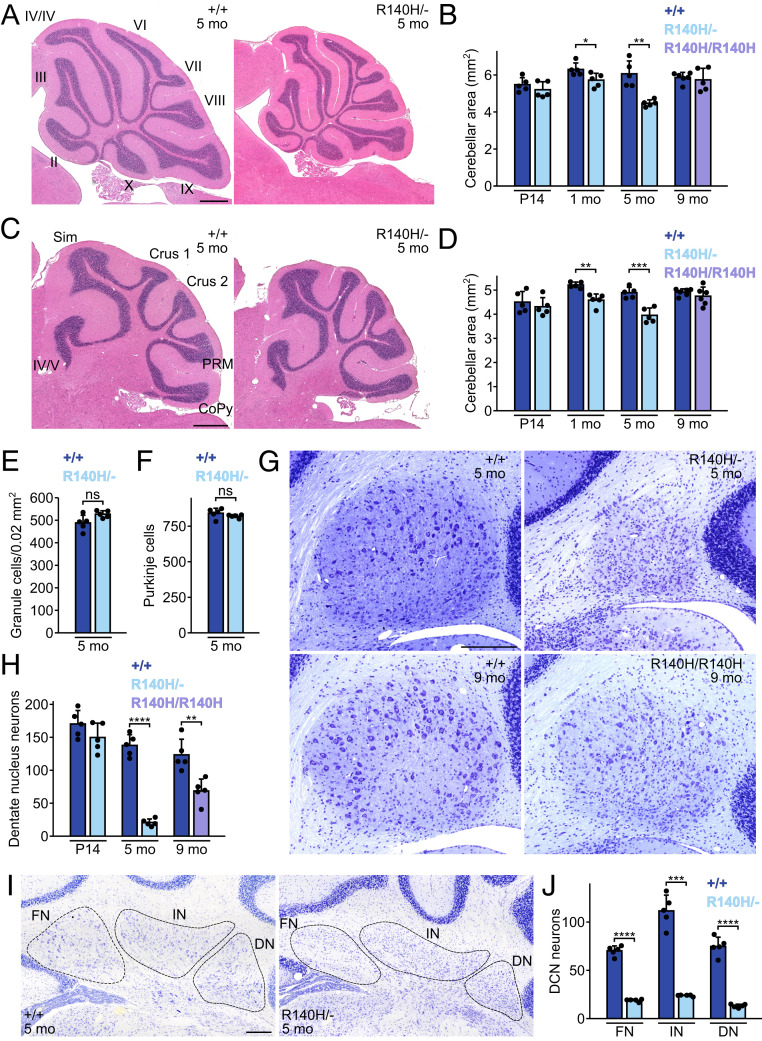
*Clp1* mutant mice have cerebellar defects. (*A* and *B*) H&E-stained midline sagittal sections of cerebellar vermis (*A*) and mean section area (*B*). (Scale bar, 500 µm.) (*C* and *D*) H&E-stained parasagittal sections of cerebellar hemispheres (*C*) and mean section area (*D*). (Scale bar, 500 µm.) (*E*) Mean granule cell density in lobule IX of vermis. (*F*) Mean Purkinje cells per midline sagittal section. (*G*) Dentate nuclei from sagittal sections stained with Cresyl violet. (Scale bar, 200 µm.) (*H*) Mean large (>100 µm^2^) dentate nucleus neurons per sagittal section. (*I*) DCN from coronal sections stained with Cresyl violet. (Scale bar, 200 µm.) (*J*) Numbers of large (>100 µm^2^) neurons per coronal hemisection in each nucleus of the DCN. Mean + SD. *t* tests with Welch’s correction. CoPy, copula pyramidis; DN, dentate nucleus; FN, fastigial nucleus; IN, interposed nucleus; PRM, paramedian lobule; Sim, simple lobule; *P ≤ 0.05; **P ≤ 0.01; ***P ≤ 0.001; ****P ≤ 0.0001; ns, not significant.

Cerebellar atrophy is observed in some *Clp1*^*R140H/R140H*^ patients, but not in *Clp1*^*K127A/K127A*^ mice (1, 2, 4). Cross-sectional areas of the vermis and the cerebellar hemispheres in *Clp1*^*R140H/−*^ mice were similar to those of wild-type mice at P14, but were decreased by 1 mo of age (Fig. 2 *A*–*D*). In contrast, aged *Clp1*^*R140H/R140H*^ mice had normal cerebellar measurements. To identify cerebellar cell types affected by *Clp1* mutation, we counted granule cells, Purkinje cells, and large DCN neurons in sections from 5-mo-old wild-type and *Clp1*^*R140H/−*^ cerebellum. We observed a trend (*P* = 0.063) toward higher granule cell density in lobule IX of *Clp1*^*R140H/−*^ mice, which suggests that any granule cell loss in mutants is low relative to the loss of cerebellar volume (Fig. 2*E*). Numbers of Purkinje cells, identified on the basis of calbindin expression, were not significantly different between genotypes (Fig. 2*F*). In contrast, large neurons in the dentate nuclei were 85% less numerous in 5-mo-old *Clp1*^*R140H/−*^ mice than in controls; however, the numbers of these neurons in P14 mice were not different between genotypes (Fig. 2 *G* and *H*). These neurons were also less abundant in 9-mo-old *Clp1*^*R140H/R140H*^ mice. In addition, loss of DCN neurons occurred in the fastigial and interposed nuclei of *Clp1*^*R140H/−*^ mice, which had 73% and 79% fewer large neurons at 5 mo, respectively (Fig. 2 *I* and *J*).

### Dysregulation of Numerous tRNA Gene Products, but Not Mature tRNAs, in *Clp1* Mutant Tissues.

Given the dysregulation of intron-containing tRNA gene products reported in the *Clp1*^*K127A/K127A*^ mouse and in PCH10 patient cells (1, 2, 4), we assayed the intron-containing tRNA families (Arg-UCU, Ile-UAU, Leu-CAA, and Tyr-GUA) in *Clp1*^*R140H/−*^ mice. Northern blots were performed at P14 on spinal cord, cerebellum, and fore-/midbrain—tissues affected in patients—and kidney, which appeared grossly normal in *Clp1*^*R140H/−*^ and *Clp1*^*K127A/K127A*^ mice (4). For each tRNA family, we separately probed for the 5′ exon, the 3′ exon, and the intron, using either a “pan” oligonucleotide or a pool of oligonucleotides to recognize all intron-containing family members (*SI Appendix*, Tables S1–S4).

**Fig. 3.**
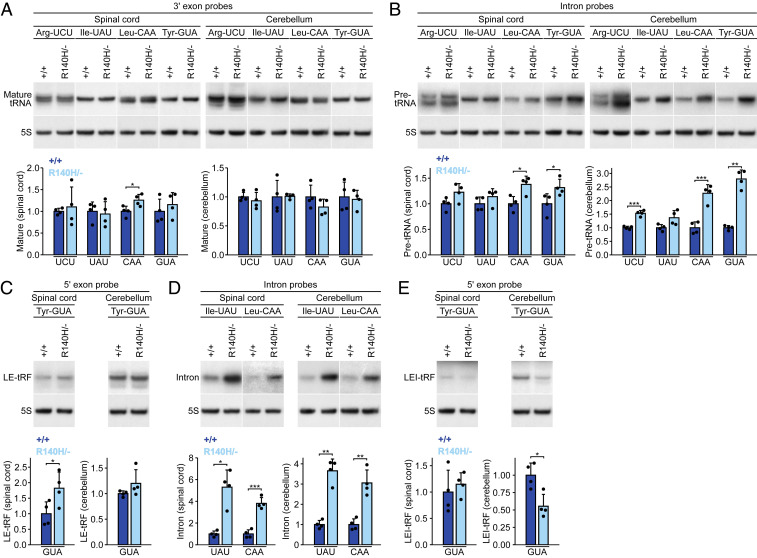
Levels of some tRNA products, but not mature tRNAs, are altered in *Clp1*^*R140H/−*^ mice. (*A*–*E*) Northern blots on spinal cord and cerebellum from P14 mice showing various tRNA products derived from the four families of intron-containing tRNA genes. Signal intensities normalized to 5S rRNA (loading control). Mean + SD. *t* tests with Welch’s correction. *P ≤ 0.05; **P ≤ 0.01; ***P ≤ 0.001.

Mature tRNAs from intron-containing genes were previously described as depleted in PCH10 patient-derived induced neurons; however, no depletion was observed in patient-derived fibroblasts or in *Clp1*^*K127A/K127A*^ mouse embryonic fibroblasts or spinal cord (1, 2, 4). In *Clp1*^*R140H/−*^ mice, the levels of mature tRNAs did not change for 13 of 16 tRNA/tissue combinations (Fig. 3*A* and *SI Appendix*, Tables S1–S4). For the other three, results were equivocal, with probes against one exon suggesting modest up-regulation while probes against the other showed no significant dysregulation; notably, down-regulation was never observed. In previous accounts of PCH10 patient cells, intron-containing pre-tRNAs were variably described as up-regulated, down-regulated, or unchanged, depending on the gene, study, and cell type (1, 2). In the *Clp1*^*R140H/−*^ mouse, pre-tRNA levels were significantly increased in 9 of 12 tRNA/neural tissue pairs, but not changed in kidney (Fig. 3*B* and *SI Appendix*, Tables S1–S4).

We also investigated levels of 5′ leader-5′ exon fragments (LE-tRFs) and 3′ exon-3′ trailer fragments (ET-tRFs). These fragments may be produced as splicing intermediates, if intron excision precedes leader/trailer processing in metazoans, and thus alterations in their levels could indicate splicing defects. Moreover, a previous study found elevated levels of Tyr-GUA, Arg-UCU, and Leu-CAA LE-tRFs in *Clp1*^*K127A/K127A*^ mouse cells (4). Up-regulation of Tyr-GUA LE-tRFs was observed in the *Clp1*^*R140H/−*^ spinal cord, but not in other mutant tissues (Fig. 3*C* and *SI Appendix*, Table S4). The only other LE-tRFs to change significantly were Arg-UCU LE-tRFs, which were up-regulated in the mutant kidney, but not in neural tissues (*SI Appendix*, Table S1). Although ET-tRFs have not been reported to vary in abundance in *CLP1* mutants, three fragments suspected to be ET-tRFs were dysregulated in *Clp1*^*R140H/−*^ tissues; Arg-UCU tRFs in kidney and two distinct Tyr-GUA ET-tRFs in the cerebellum and kidney were all up-regulated (*SI Appendix*, Tables S1 and S4).

Alterations in levels of free exons have not been previously reported in *Clp1* mutants. In our experiments, free exons were rarely detected, suggesting that intron excision may typically precede removal of the leader and trailer. Free 3′ exons were not sufficiently abundant to be quantified and 5′ exons were only quantifiable in a quarter of the tissue/tRNA family combinations examined. The Arg-UCU 5′ exon was down-regulated in the fore-/midbrain, but no other exons changed in abundance (*SI Appendix*, Table S1).

We next evaluated levels of free introns, some of which were elevated in *CLP1*^*R140H/R140H*^ patient fibroblasts (1, 2). We were only able to detect introns in the Ile-UAU and Leu-CAA families, likely due to technical reasons (Arg-UCU introns are unusually short and Tyr-GUA introns are especially variable in length). For each family, we observed two bands likely corresponding to introns. Quantification of both bands together or the darker band alone (the darker band constituted 71 to 94% of total intronic signal, while the lighter band was not detected in all tissues) revealed that *Clp1*^*R140H/−*^ mice had significant up-regulation of Ile-UAU and Leu-CAA introns in all four tissues tested (Fig. 3*D* and *SI Appendix*, Tables S2 and S3).

It is unclear whether additional tRFs are also dysregulated in *Clp1* mutants. Thus, we quantified the remaining tRFs and identified several that differed in abundance between genotypes. Fragments corresponding to the leader, 5′ exon, and intron (LEI-tRFs) of Tyr-GUA and Arg-UCU families were down-regulated in various *Clp1*^*R140H/−*^ tissues (Fig. 3*E* and *SI Appendix*, Tables S1 and S4). In addition, apparent Ile-UAU 5′ exon-intron tRFs were up-regulated in the mutant spinal cord and cerebellum (*SI Appendix*, Table S2). We also observed up-regulation of several medium-to-large fragments of unknown composition that were very low in abundance and recognized only by intronic probes (*SI Appendix*, Fig. S2).

Having systematically analyzed alterations in tRNA gene products in *Clp1*^*R140H/−*^ tissues at P14, we next assessed the spinal cord and cerebellum of *Clp1*^*R140H/R140H*^ mice at P14 and 9 mo of age. Mature Leu-CAA tRNA levels were normal in both *Clp1*^*R140H/R140H*^ tissues at both ages (*SI Appendix*, Fig. S3*A*). Levels of Leu-CAA pre-tRNA, which were elevated in both spinal cord and cerebellum of *Clp1*^*R140H/−*^ mice at P14, were normal in R140H homozygotes at P14 (*SI Appendix*, Fig. S3*B*). At 9 mo, however, *Clp1*^*R140H/R140H*^ mice had higher levels of this pre-tRNA than wild-type mice. Levels of Tyr-GUA LE-tRFs followed the same trends observed in *Clp1*^*R140H/−*^ mice, with up-regulation in the spinal cord but no change in cerebellum at P14 (*SI Appendix*, Fig. S3*C*). At 9 mo, levels of Tyr-GUA LE-tRFs did not differ significantly between wild-type and *Clp1*^*R140H/R140H*^ mice in either tissue. Leu-CAA intron levels were elevated in *Clp1*^*R140H/R140H*^ mice in all four combinations of ages and tissues (*SI Appendix*, Fig. S3*D*). Down-regulation of Tyr-GUA LEI-tRFs, which was observed in *Clp1*^*R140H/−*^ cerebellum, was also seen in P14 *Clp1*^*R140H/R140H*^ mice, although in this case spinal cord levels were significantly down-regulated while cerebellar levels only trended in that direction (63% of wild-type levels, *P* = 0.053) (*SI Appendix*, Fig. S3*E*). No changes in Tyr-GUA LEI-tRFs were observed between genotypes at 9 mo.

To determine whether changes in pre-tRNAs and tRFs are solely associated with intron-containing genes, we probed cerebellar RNA from P14 wild-type and *Clp1*^*R140H/−*^ mice with pan-family probes for three intronless tRNA families: Arg-CCU, Ile-AAU, and Leu-CAG. We did not observe changes in mature tRNA levels for any of the three families (*SI Appendix*, Fig. S4*A*). However, we did see up-regulation of one of three Arg-CCU pre-tRNAs and of Arg-CCU tRFs in the mutant cerebellum (*SI Appendix*, Fig. S4 *B* and *C*). These data demonstrate that up-regulation of pre-tRNAs and tRFs is not restricted to intron-containing genes, raising the question of whether tRNA dysregulation in *Clp1* mutants should be uniformly attributed to defects in tRNA splicing.

### Increased Distal PAS Usage in *Clp1*^*R140H/−*^ Spinal Cord.

**Fig. 4.**
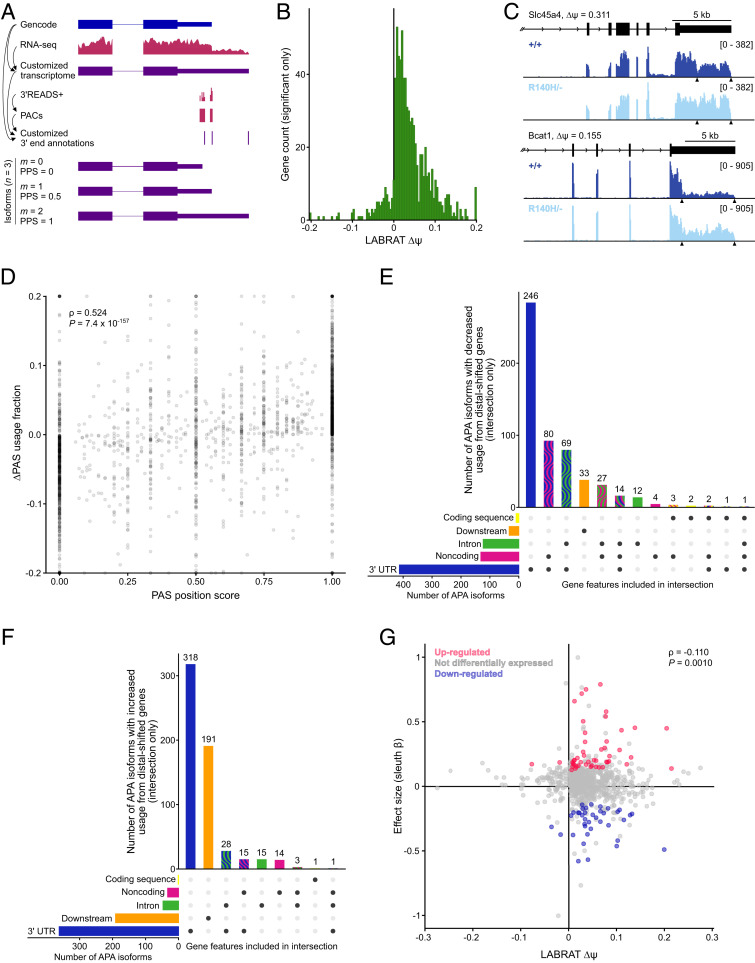
PAS selection and gene expression are perturbed in P14 *Clp1*^*R140H/−*^ spinal cord. (*A*, *Upper*) Workflow for annotation of PACs. A customized reference transcriptome was extrapolated by comparing reference (blue) and experimental (pink) data. (*Lower*) APA isoforms inferred from the customized reference data. For each gene, APA isoforms are assigned sequential values *m* corresponding to their rank relative to other APA isoforms, with the APA isoform derived from the use of the most proximal PAS set at *m* = 0 and the APA isoform from the most distal PAS set at *m* = *n* − 1, where *n* is the total number of APA isoforms for the gene. The PAS position score (PPS) is defined as *m* ÷ (*n* − 1). (*B*) Histogram of genes with significant alternative PAS usage in *Clp1*^*R140H/−*^ spinal cord. LABRAT ∆ψ = *Clp1*^*R140H/−*^ ψ − *Clp1*^*+/+*^ ψ. ∆ψ values were clipped at ±0.2. (*C*) Read counts from representative RNA-seq libraries showing the 3′ ends of example genes with significant distal shifts in PAS usage in *Clp1*^*R140H/−*^ spinal cord. Reference sequences (mm10) are shown in black and PASs are indicated by arrowheads. (*D*) Relationship of PAS usage to PAS position for all APA isoforms from genes in which ∆ψ ≠ 0 and *P*-adjusted ≤ 0.05. PAS usage fraction is the proportion of reads attributed to a given APA isoform relative to the total number of reads associated with the gene. ∆PAS usage fraction = *Clp1*^*R140H/−*^ PAS usage fraction − *Clp1*^*+/+*^ PAS usage fraction. Correlation was assessed by Spearman’s rank correlation. Values for ∆PAS usage fraction were clipped at ±0.2. (*E* and *F*) UpSet plots showing the numbers of gene features that overlap with the PASs of differentially utilized APA isoforms (∆PAS usage fraction ≠ 0 and *P*-adjusted ≤ 0.05) from genes with differential PAS usage (LABRAT ∆ψ ≠ 0 and *P*-adjusted ≤ 0.05). APA isoforms that were derived from distal-shifted genes and show decreased usage in the mutant (*E*) and APA isoforms that were derived from distal-shifted genes and show increased usage in the mutant (*F*). (*G*) Relationship of differential expression to PAS usage for genes with altered PAS usage in *Clp1*^*R140H/−*^ spinal cord. Genes in which ∆ψ ≠ 0 and *P*-adjusted ≤ 0.05 are shown. sleuth β is an approximation of the natural log of the fold-change of expression in *Clp1*^*R140H/−*^ spinal cord relative to wild-type spinal cord. Values were clipped at *x* = ±0.275 and *y* = ±1. Correlation was assessed by Spearman’s rank correlation. kb, kilobases.

PAS selection is perturbed in several cell types upon knockdown of *CLP1*, but whether the *Clp1* R140H mutation alters PAS usage is not known. To address this question, we made standard poly(A)-selected RNA-sequencing (RNA-seq) libraries from the spinal cords of P14 wild-type and *Clp1*^*R140H/−*^ mice. We first compared our RNA-seq data to the Gencode reference transcriptome, using StringTie to assemble a customized transcriptome such that the 3′ ends observed in our RNA-seq data were reflected in our reference transcriptome (Fig. 4*A*). To identify unannotated PASs, we generated 3′READS+ libraries (20) representing the 3′ ends of transcripts from GFP-expressing motor neurons from neonatal Hlxb9-GFP and Hlxb9-GFP; *Clp1*^*R140H/−*^ spinal cords. While these libraries had too few unique reads to reveal differences in gene expression or PAS usage on their own, they were sufficient to annotate the locations of PAS clusters (PACs) (Dataset S1). These PACs, along with PASs inferred from the 3′ ends of the Gencode and customized reference transcripts, were combined to generate a customized annotation of gene ends from which possible alternative polyadenylation (APA) isoforms were inferred (Fig. 4*A*). The relative usage of these APA isoforms in our RNA-seq libraries was analyzed using lightweight alignment-based reckoning of alternative three-prime ends (LABRAT) (21), which generated a ψ score (ranging from 0 to 1) for each gene in each genotype. Lower ψ values indicate preferential use of proximal PASs and higher values indicate preferential use of distal PASs. For each gene, the ∆ψ score was computed by subtracting the wild-type ψ from that of the mutant. PAS usage was altered (i.e., *P*-adjusted ≤ 0.05) in 797 genes; of these, 712 (89%) had a positive ∆ψ, which indicates a strong bias toward use of distal sites in the *Clp1*^*R140H/−*^ spinal cord (Fig. 4 *B* and *C* and Dataset S2).

We next evaluated differential PAS usage on the level of individual APA isoforms. For every gene in a library, LABRAT determined which APA isoforms were present and the expression level of each. From this, we calculated the fractional contribution of each to total gene levels (PAS usage fraction) in order to identify individual PASs with altered usage (Dataset S3). APA isoforms were classified as having increased, decreased, or unchanged PAS usage fractions in the *Clp1*^*R140H/−*^ relative to the wild-type spinal cord, and further subdivided based on the position of the PAS used by that APA isoform relative to the other PASs in the gene (*SI Appendix*, Fig. S5*A*). We found that a majority of APA isoforms with increased PAS usage fractions in mutant mice were associated with the most distal PAS of their gene, and very few were associated with the most proximal site. Conversely, the largest number of the APA isoforms with decreased PAS usage fractions in mutant mice came from use of the proximal PAS, with far fewer associated with the most distal site. We confirmed this shift in PAS usage by examining all APA isoforms from genes with differential PAS usage between wild-type and mutant spinal cord. For each APA isoform, we subtracted the PAS usage fraction of wild-type mice from that of *Clp1*^*R140H/−*^ mice. This difference was expressed as a function of the PAS position score, defined as *m* ÷ (*n* – 1), where *n* is the total number of APA isoforms for the gene and *m* is the position of an APA isoform relative to others from the same gene, with *m* set to 0 for the most proximal and *m* = *n* – 1 for the most distal (Fig. 4 *A* and *D*). This analysis demonstrated that the usage of PASs between the proximal and distal PASs increases in the mutant spinal cord as these sites become more distal. Together, these data suggest that CLP1 promotes selection of or cleavage at proximal PASs, and that this function is impaired by the R140H mutation.

Because increased or decreased use of a PAS in the coding sequence/intron would be predicted to reduce or elevate levels of full-length protein, respectively, we checked which gene features overlapped with differentially utilized PASs (Dataset S4). We focused on APA isoforms for which both APA isoform-level and gene-level analyses demonstrated altered PAS usage. We found that very few PASs were categorized as occurring solely in the coding sequence and/or intron and not in a 3′ UTR and/or a noncoding transcript (16 of 551 and 18 of 636 APA isoforms used less and more frequently in mutants, respectively) (Fig. 4 *E* and *F* and *SI Appendix*, S5 *B* and *C*). This suggests that changes in PAS usage in the mutant rarely alter the coding sequence. To verify that intronic PAS usage was infrequently altered in mutants, we used an independent strategy to specifically assess use of intronic PASs. This showed that only 14 of 23,576 intronic sites were differentially utilized (Dataset S5).

We then asked whether closely spaced genes were enriched among the genes with distal-shifted PAS usage in the *Clp1* mutant. Closely spaced genes are bound by high levels of PCF11, and are down-regulated in *PCF11*-knockdown cells, suggesting that they are particularly reliant upon CFII (18, 19). Indeed, relative to genes with unchanged PAS usage in the mutant, the genes that favored distal PASs tended to be closer to their 10 nearest expressed/polyadenylated neighbors than genes with unchanged PAS usage (*SI Appendix*, Fig. S5*D*). Similarly, short genes were previously reported to be down-regulated in *Pcf11*-knockdown cells (18), and we observed that genes that favored distal PASs in *Clp1* mutants also tended to be shorter (*SI Appendix*, Fig. S5*E*). These data suggest that CLP1, like PCF11, is especially important for proper PAS selection in densely packed and short genes.

We next analyzed genes with proximal- or distal-shifted PAS usage in the *Clp1*^*R140H/−*^ spinal cord in terms of gene ontology and predicted subcellular localization. Gene ontology analyses of biological processes, cellular components, and molecular functions produced no significant terms for any of these categories. We then asked whether the changes in PAS selection would be predicted to influence transcript localization. A previous study found that for a number of genes, PAS selection correlated with subcellular localization in neurons; in particular, there were many genes with alternative last exons (ALEs) for which the fractional contribution of the distal ALE relative to the proximal ALE was higher in neurites than in the soma (22). Our LABRAT analysis evaluated ALE usage in these genes, finding that 4 had increased proximal PAS usage in the mutant and 19 had increased distal PAS usage (Dataset S2). Therefore, it seems likely that altered PAS selection in *Clp1* mutants could change the localization of a subset of transcripts, primarily promoting a shift from somatic to neuritic localization.

Next, we investigated whether altered PAS usage might change the susceptibility of genes to regulation by microRNAs. First, we looked for cases in which microRNA binding sites were included in a higher fraction of transcripts in mutant relative to wild-type spinal cord by searching for predicted microRNA binding sites within the sequences between proximal and distal PASs in genes with increased distal PAS usage in the mutant. Of 518 distal-shifted genes for which we could identify at least one specific distal PAS with increased usage in the mutant, 451 contained at least one predicted microRNA binding site (Dataset S6). In total, we observed 4,342 target/microRNA pairs representing 1,367 unique microRNAs. Of the 1,367 microRNAs, 249 are known to be expressed in spinal cord neurons (23). Of these, each targeted no more than 17 of the distal-shifted genes. Second, we performed the converse analysis, assessing sequences between proximal and distal PASs in genes with decreased distal PAS usage in the mutant. Thirty-seven of 44 proximal-shifted genes for which we could identify at least one specific distal PAS with decreased usage in the mutant contained at least one predicted microRNA binding site (Dataset S6). We observed 331 target/microRNA pairs, representing 276 unique microRNAs. Of these, 42 microRNAs, each with one to four targets, are known to be expressed in spinal cord neurons (23). Finally, we looked for enrichment of microRNA binding sites in the sequences of interest in either distal-shifted or proximal-shifted genes compared to a background consisting of the 3′ UTRs of all expressed genes that were not distal- or proximal-shifted, respectively. We found no microRNAs enriched in sequences from either group.

### Gene Biotype, Density, and Length Correlate with Differential Gene Expression.

Next, we analyzed differential gene expression, identifying 845 up-regulated and 418 down-regulated genes in the P14 mutant spinal cord (*SI Appendix*, Fig. S6*A* and Dataset S7). Gene ontology analysis of up-regulated genes produced several terms pertaining to mRNA translation—primarily due to the modest up-regulation of >30 ribosomal protein genes—while down-regulated genes were associated with terms related to mitochondria (Dataset S8). Inspection of the differentially regulated genes revealed that the up-regulated gene set was enriched with protein-coding genes and depleted of noncoding genes, while down-regulated and unchanged genes had similar proportions of most biotypes (*SI Appendix*, Fig. S6*B*). Assessment of the relative expression of all the genes in each of several biotypes revealed that protein-coding genes as a group were up-regulated in the mutant spinal cord, and that major categories of noncoding genes—antisense, long intergenic noncoding RNA, and processed pseudogenes—were down-regulated (*SI Appendix*, Fig. S6*C*).

Given that short and closely spaced genes were prevalent among the genes with increased distal PAS usage in the mutant spinal cord, which we suspected might indicate that they were especially dependent upon CLP1 for 3′ end processing, we hypothesized that these genes might experience high rates of cleavage failure in the *Clp1* mutant. This would be represented in our RNA-seq libraries by lower expression of short and closely spaced genes, as was observed in *PCF11*-knockdown cells (18, 19). We therefore evaluated the relationship between gene density and gene expression by graphing the relative expression of each gene as a function of the average distance between that gene and each of its 10 nearest expressed and polyadenylated neighbors (as identified by 3′READS+) (*SI Appendix*, Fig. S7*A*). This showed a strong positive correlation, reflecting down-regulation of closely spaced genes and up-regulation of isolated ones. Down-regulation of densely packed genes could indicate transcriptional interference, a phenomenon in which readthrough transcription at one gene interferes with the transcription of a neighboring gene toward which it is transcribed. However, while genes down-regulated in the *Clp1*^*R140H/−*^ spinal cord were closer to their neighbors than were up-regulated genes, changes in gene expression were independent of the position or orientation of the neighbor, which suggests that if transcriptional interference is contributing to the down-regulation of closely spaced genes, its effects are not limited to adjacent genes (*SI Appendix*, Fig. S7*B*).

Similarly, we observed that gene length correlated with up-regulation in mutants (*SI Appendix*, Fig. S7*C*). Furthermore, genes down-regulated in mutants were significantly shorter than unchanged genes, while up-regulated genes were significantly longer (*SI Appendix*, Fig. S7*D*). Because we had observed that gene biotype was associated with relative expression in the mutant, and noncoding RNAs tend to be shorter than protein-coding RNAs, we examined the relationship between gene length and relative expression in noncoding RNAs and protein-coding RNAs independently (*SI Appendix*, Fig. S7 *E* and *F*). Although the correlations were weaker within each of these groups alone than within the combined group (*SI Appendix*, Fig. S7*C*), both were statistically significant.

To examine the possibility that some of the differential expression in the *Clp1*^*R140H/−*^ spinal cord could be correlated with altered PAS selection, we examined the relative expression of genes with altered PAS usage as a function of LABRAT ∆ψ. We observed little correlation between ∆ψ and differential expression (sleuth β) (Fig. 4*G* and Dataset S9). Comparison of genes with distal-shifted PAS usage with up- and down-regulated genes showed that genes with increased distal PAS usage were more likely than other expressed genes to be differentially expressed; of the 796 Gencode reference genes associated with the 712 customized reference genes with increased distal PAS usage, 46 were up-regulated (*P* = 2.5 × 10^−4^ by Fisher’s exact test) and 36 were down-regulated (*P* = 1.1 × 10^−7^). In contrast, genes with proximal-shifted PAS usage were not more likely than other genes to be differentially expressed (two up-regulated genes, *P* = 0.84; two down-regulated genes, *P* = 0.64).

We next explored whether altered PAS usage could impact differential expression by increasing or decreasing the susceptibility of transcripts to destabilization by microRNAs. However, protein-coding distal-shifted genes with predicted microRNA binding sites had expression levels comparable to those of other protein-coding genes (distal-shifted, *P* = 0.14). Similarly, protein-coding proximal-shifted genes with predicted microRNA binding sites had expression levels comparable to those of other protein-coding genes (proximal-shifted, *P* = 0.21). In distal-shifted genes, microRNA binding sites were present in similar fractions of down-regulated (67%) and up-regulated (65%) genes. Together, these data suggest that the presence of predicted microRNA binding sites does not correlate with gene down-regulation.

Finally, we examined whether the PASs used differentially between the wild-type and the *Clp1*^*R140H/−*^ spinal cord were associated with different nucleotide motifs (*SI Appendix*, Table S5). Aside from permutations of the canonical cleavage and polyadenylation specificity factor (CPSF) binding site, we failed to find motifs enriched in more than a modest fraction of sequences. We also looked for motif enrichment around the PASs of up-regulated and down-regulated genes, and identified seven and eight motifs, respectively.

In summary, mutation of *Clp1* is associated with a shift from proximal to distal PASs. The changes in PAS selection observed in mutants were predicted to often alter the susceptibility of transcripts to regulation by microRNAs, and to occasionally change the coding sequence or subcellular localization. Altered PAS usage was particularly common at closely spaced and short genes, and these, along with noncoding genes, were expressed at lower levels in mutant than in wild-type mice. We also observed many changes in gene expression in mutant mice, but whether these changes are directly related to altered PAS selection is unclear. Collectively, these data suggest that wild-type CLP1 is required for normal PAS selection and gene expression.

## Discussion

In this study, we present *Clp1*^*R140H/−*^ and *Clp1*^*R140H/R140H*^ mice as models of PCH10. Both models replicate the motor defects and cerebellar atrophy observed in patients, and confirm the previously suspected motor neuron death. These models also unveil other phenotypes, with death of nonmotor neurons in the spinal cord and DCN neurons.

We profiled products derived from intron-containing tRNA genes in *Clp1*^*R140H/−*^ and *Clp1*^*R140H/R140H*^ mice to illuminate the role of CLP1 in the TSEN complex and to identify any systematic changes in tRNA metabolism that might underlie pathogenesis. Several roles for CLP1 in tRNA metabolism have been proposed. Studies examining cleavage of an exogenous pre-tRNA in *Clp1* mutant extracts suggested that CLP1 promotes tRNA cleavage (1, 4). If cleavage were impaired in *Clp1* mutant tissues, we would normally expect levels of mature tRNAs to be diminished. However, a PCH2-associated mutation in TSEN54 (A307S) that reduces the cohesiveness of the TSEN complex did not change mature tRNA levels, but instead caused higher levels of pre-tRNAs, suggesting that homeostatic up-regulation of tRNA transcription might compensate for the reduced efficiency of tRNA cleavage (24). Like *TSEN54* mutant cells, *Clp1* mutant neural tissues had up-regulation of pre-tRNAs but no change in mature tRNA levels. However, pre-tRNA up-regulation may not be directly linked to diminished splicing of that tRNA: levels of intronless pre–Arg-CCU tRNAs were also increased in mutant tissues. This result suggests that either diminished pre-tRNA cleavage has a larger effect on pre-tRNA transcription than anticipated, or at least some of the observed up-regulation of pre-tRNAs is independent of impaired tRNA splicing.

CLP1 has also been hypothesized to directly affect tRNA ligation, with one study concluding that it might impede the canonical ligation mechanism, but another suggesting it could promote ligation through a noncanonical pathway (5, 9). While we did observe several up-regulated LE- and ET-tRFs in *Clp1* mutant tissues, dysregulation of both LE- and ET-tRFs from the same tRNA family was only observed for 1 of 16 tissue/tRNA family combinations (Arg-UCU in kidney). Similarly, only one free exon was dysregulated in *Clp1*^*R140H/−*^ tissues. Thus, our data fail to support the hypothesis that tRNA ligation is altered in *Clp1* mutant mice, although it remains possible that concomitant defects in tRNA cleavage and ligation could obscure each other.

In addition, CLP1 has also been hypothesized to impact processing of free introns after excision from the pre-tRNA. In yeast, intron degradation by the 5′-to-3′ exonuclease Xrn1 requires prior phosphorylation of the 5′-OH of the intron (11). CLP1 has been shown to phosphorylate free tRNA introns in human cell extracts (5), raising the possibility that degradation of tRNA introns in metazoans requires CLP1 (11). Consistent with this hypothesis, levels of several introns were higher in PCH10 patient cells (1). Intron phosphorylation by CLP1 has also been hypothesized to prevent intron circularization in metazoans (9). Overexpression of wild-type CLP1, but not kinase-deficient K127A CLP1, diminished circularization of introns by RTCB in human cells, while levels of circularized introns were increased by knockdown of the *Drosophila CLP1* homolog *cbc*. In *Clp1*^*R140H/−*^ tissues, we observed up-regulation of tRNA products of the expected electrophoretic mobilities of both linear and circular introns. Introns that ran true to size and thus were likely linear made up a majority of free intronic signal, and were always up-regulated in mutant tissues (Fig. 3*D* and *SI Appendix*, Tables S2 and S3). The faster-migrating bands, which may correspond to circularized introns, were only observed in three *Clp1*^*R140H/−*^ tRNA family/tissue combinations, and were up-regulated in two (*SI Appendix*, Tables S2 and S3). Thus, our data support the hypothesis that CLP1 phosphorylates metazoan introns to prepare them for exonucleolytic degradation, as occurs in yeast, but do not rule out a minor role for CLP1 in down-regulating intron circularization.

Previous research has hypothesized that pathogenesis in PCH10 stems from dysregulation of tRNA metabolism (1, 2, 4). This hypothesis is particularly attractive because the four *Tsen* genes, in addition to encoding proteins that form a complex with CLP1, are each mutated in a subset of PCH cases (25, 26). Previous studies have shown that altered levels of tRNA gene products can initiate signaling cascades that affect cell survival (27–29). In one putative mechanism, Tyr-GUA LE-tRFs are hypothesized to sequester pyruvate kinase M2 in *Clp1* mutant mice and zebrafish, potentially leading to p53 (TRP53) activation and cell death (4, 30). However, while K127A homozygous mice had substantial up-regulation of Tyr-GUA LE-tRFs in several tissues (4), these fragments were only modestly up-regulated in the spinal cord of P14 *Clp1*^*R140H/−*^ mice and not up-regulated in the cerebellum. Furthermore, levels of these fragments in mutant spinal cord were not significantly elevated relative to controls at 9 mo of age, when motor neuron death is ongoing. Our data therefore cast doubt on the hypothesis that these tRFs are instrumental in cell death. Pre-tRNAs could present a more promising link to pathogenesis, given that their up-regulation in *Clp1*^*R140H/−*^ and *Clp1*^*R140H/R140H*^ mice is limited to affected tissues. However, a previous study in which pre-tRNAs were overexpressed in induced neurons found no effects on cell viability (2), which implies that while elevated pre-tRNA levels may be biomarkers of PCH10, they are probably not directly responsible for neurodegeneration in PCH10 patients. Finally, elevated levels of free introns may promote cell death in neurons (11); however, intron levels were increased in the *Clp1*^*R140H/−*^ kidney, as well as in affected neural tissues, and a previous study showed that intron overexpression was not toxic in a human neuroblastoma cell line (30). We conclude that *Clp1* mutants have altered tRNA metabolism, but whether this contributes to neuronal death is unclear.

In addition to profiling tRNA gene products, we also analyzed PAS usage and gene expression in *Clp1*^*R140H/−*^ spinal cord. Our analyses were informed by previous studies showing that PCF11, which together with CLP1 composes CFII of the cleavage/polyadenylation complex, promotes mRNA 3′ processing (18, 19). Strong parallels between our work and observations from *PCF11*-knockdown cells suggest that CLP1 and PCF11 function together to cleave newly transcribed pre-mRNAs of short, closely spaced genes. As we observed in the *Clp1*^*R140H/−*^ spinal cord, *PCF11*-knockdown cells exhibited down-regulation of both short and closely spaced genes (18, 19). In addition, greater enrichment of PCF11 was observed at the 3′ ends of closely spaced genes relative to isolated genes in wild-type cells (19). Whether gene length, density, or another property correlating with length and density, such as gene biotype, directly causes, or is simply correlated with, transcriptional down-regulation in *Clp1* mutant and *PCF11*-knockdown cells is unclear. The preference of CLP1 for proximal PASs relative to distal PASs and for short genes relative to long genes could be due to the interaction of CLP1 with members of the cap-binding complex, which binds the 7-methylguanosine cap at the 5′ end of an mRNA (31). CLP1 is associated with cap-binding complex members ARS2 and CBP20, and knockdown of *ARS2*, *CBP20*, or *CLP1* caused readthrough transcription on short reporter genes in human cells. However, in *ARS2*- and *CBP20*-knockdown cells, cleavage of longer reporters was not impaired. Although elongated reporters were not assayed in *CLP1*-knockdown cells, this implies that the distance between the 5′ cap and the PAS might impact recruitment of CLP1, and thereby change the mechanism by which cleavage occurs.

Knockdown of *PCF11* caused decreased use of intronic PASs, resulting in elevated transcript levels of long genes, which frequently harbor such PASs (18, 19). This phenomenon has also been reported in *CLP1*-knockdown cells, although the prevalence of decreased intronic PAS usage in these cells was not clear (16). However, we did not observe widespread alterations in intronic PAS usage in *Clp1*^*R140H/−*^ spinal cord despite seeing up-regulation of long genes, which suggests that CLP1 does not contribute substantially to selection of intronic PASs, or that its contribution is not significantly impaired by the R140H mutation.

Our data suggest that impairment of mRNA cleavage in the *Clp1*^*R140H/−*^ spinal cord causes a shift toward distal PAS usage and may impede the maturation of a subset of transcripts. Could this impairment of mRNA processing underlie the pathogenicity of *Clp1* mutations? Reduced mRNA maturation is predicted to diminish the abundance of transcripts. Indeed, five of the mitochondrial genes down-regulated in mutants have orthologs associated with neurological disease in *Drosophila* (*Miga2*) or humans (*Apoo*, *Ndufaf3*, *Ndufaf8*, and *Pet100*) (32–36). In addition, because PAS selection impacts transcript stability, localization, and translation efficiency, as well as localization of the encoded protein, the alteration of PAS usage in *Clp1*^*R140H/−*^ mice likely changes gene function (12). Furthermore, *clp1*^*−/−*^ and *pcf11*^*−/−*^ zebrafish have similar phenotypes, dying by 4 d postfertilization with extensive CNS cell death, which suggests that these mutants could share a common pathogenic mechanism (2, 19). Together, our data suggest that the neuronal death associated with the *Clp1* R140H mutation may be related to the role of CLP1 in mRNA 3′ processing.

## Materials and Methods

### Mice.

*Clp1* (c.419G > A and c.414–429∆) mutant mice were generated by pronuclear injection of Cas9 mRNA, guide RNA, and a donor DNA oligo into C57BL/6J zygotes. The guide RNA corresponded to *Clp1* c.403–422, and was accompanied by a donor oligo corresponding to *Clp1* c.357–486 introducing the c.419G > A mutation (nucleotide positions are numbered based on the coding sequence of NM_133840.2). Injected zygotes were implanted into pseudopregnant females, and the resulting pups underwent sequencing of the complete *Clp1* coding sequence. Founder mice were back-crossed to C57BL/6J mice for at least two generations prior to intercrossing. *Clp1* mutant mice have been deposited with The Jackson Laboratory as stock numbers 036709 (R140H) and 036710 (null). B6.Cg-Tg(Hlxb9-GFP)1Tmj/J mice (Jackson Laboratory, stock no. 005029) were obtained from The Jackson Laboratory. The Jackson Laboratory Animal Care and Use Committee and the University of California San Diego Animal Care and Use Committee approved mouse protocols. Mice were group housed with a 12-h light/12-h dark cycle. Mice of both sexes were used for each experiment.

*Clp1* mice were genotyped by PCR using the following primers: Clp1-WT-forward (GCT​ACT​CAA​CTA​CGC​AGT​GGG); Clp1-common-reverse (GCC​TGG​ATG​GAG​AAA​CCT​TC); Clp1-R140H-forward (ACT​CAA​CTA​CGC​AGT​GCA​TAT); Clp1-∆-forward (GAA​GAG​AGA​GGT​CCC​CGA​GT) and Clp1-∆-reverse (GGT​ACC​AGG​GAT​GGA​CAC​AG).

### Wire-Hang Test.

At the beginning of each trial, the mouse was placed on the wire-hang apparatus (37) at the −45° position of the loop, facing left. Healthy mice consistently grabbed the wire with all four paws, and walked along it, causing the wire to rotate and keeping the mouse at or near the bottom of the loop. Each mouse underwent three trials of up to 10 min each, with a minimum of 15 min of rest between successive trials. The latencies to fall were recorded and averaged across trials.

### Treadmill Test.

Each mouse was placed on a treadmill running at 10 cm/s and then videotaped from underneath the treadmill for 20 s at a frame rate of 100 frames per second by GaitScan software. We quantified the number of frames that the mouse spent performing each of four activities: walking (facing forward, with no more than the tail touching the bumper), sliding (facing forward, pressed against the bumper), facing backward, or attempting to climb the walls. If the mouse spent less than 75% of the video either walking or sliding, the test was repeated. We calculated the walking percentage as the frames spent walking divided by the sum of the frames spent walking and the frames spent sliding.

### Rotarod Test.

Mice walked on an Ugo Basile rotarod (model 7650) as it accelerated from 4 rpm to 40 rpm and the latency to fall was recorded up to a maximum duration of 10 min. Three trials with a minimum of 15 min of rest between trials were averaged.

### Histology and Immunohistochemistry.

Mice were transcardially perfused with 4% PFA in PBS (immunofluorescence) or with Bouin’s fixative (calbindin immunohistochemistry and histology), and dissected tissues were postfixed overnight. For analysis of coronal sections of cerebellum, freshly dissected tissues were drop-fixed in Bouin’s fixative. Tissues were embedded in paraffin and cut into 7-µm sections. H&E and Cresyl violet staining were performed according to standard protocols.

For ChAT and NeuN immunofluorescence, antigen retrieval was performed by microwaving sections in antigen retrieval buffer (10 mM sodium citrate with 0.05% Tween-20, pH 6.0) until boiling, followed by cooling at room temperature for 30 min. Sections were incubated with primary antibodies (ChAT, Abcam ab178850: 1:1,000; NeuN, Millipore MAB377: 1:1,000) at 4 °C overnight followed by secondary antibodies at 1:500 (goat α-rabbit IgG Alexa Fluor 555; goat α-mouse IgG1 Alexa Fluor 488) and DAPI. Calbindin immunohistochemistry was performed as described previously (38).

### Image Analysis.

Motor neurons and NeuN^*+*^ neurons were counted in sections of C6 cervical spinal cord. To quantify motor neurons, we counted ChAT-expressing cells in the anterior horns of 20 to 30 hemisections within 245 µm. To quantify NeuN-expressing cells, we counted cells in six hemisections within 245 µm. Quantifications of cerebellar neurons and the cross-sectional area of the vermis were performed on three sections taken at midline and spaced at least 14 µm apart. Matched sections (three spaced at least 7 µm apart) were used to measure the area of the cerebellar hemispheres in mutant and wild-type mice. Granule cells, visualized by hematoxylin staining, were counted within a 0.02-mm^2^ area in lobule IX at midline. Large DCN neurons were defined as Cresyl violet-stained cells with areas of >100 µm^2^ (39). For each experiment, at least five mice were analyzed per genotype. All analyses were performed blinded to genotype.

### Northern Blot Analysis.

RNA was extracted from tissues using TRIzol (Thermo Fisher). Northern blotting was performed as previously described (40). Probes and hybridization and wash temperatures are described in *SI Appendix*, Table S6.

### 3′READS+ Library Construction.

Spinal cords of P0/P1 Hlxb9-GFP and *Clp1*^*R140H/*^*^−^*; Hlxb9-GFP mice were dissociated and lightly fixed in preparation for cell sorting using a protocol adapted from previous publications (*SI Appendix*, *Supplementary Text*) (41, 42). RNA samples were pooled so that the input for each library consisted of 175 ng of RNA derived from six spinal cords. Two wild-type and two compound heterozygote 3′READS+ libraries were prepared as described (20, 43), with some modifications: RNase III fragmentation was reduced to 5 min and adapter and primer sequences came from the NEXTFLEX Small RNA-Seq Kit v3 (Bioo Scientific); 100-nt paired-end reads were generated on a HiSeq 4000.

### RNA-Seq Library Construction.

RNA was prepared from individual spinal cords from P14 wild-type and *Clp1*^*R140H/−*^ mice using TRIzol and miRNeasy Mini kit columns (Qiagen) with on-column DNase digestion. Three RNA-seq libraries per genotype were made with the Kapa Stranded mRNA-Seq Kit in accordance with the manufacturer’s instructions; 100-nt paired-end reads were generated on a NovaSeq 6000.

### Analysis of Sequencing Data.

The 3′READS+ reads were deduplicated, trimmed, and clustered to identify PACs. RNA-seq reads were trimmed and pseudoaligned to the transcriptome, and differential expression was analyzed using sleuth (44). Customized reference transcripts and PACs were used by LABRAT to analyze PAS usage (21). Detailed methods are provided in the *SI Appendix*, *Supplementary Text*.

### Statistics.

Student’s *t* tests with Welch’s correction and one-way ANOVA with Tukey’s multiple comparisons tests were used to analyze weights, motor tests, cell counts, cerebellar measurements, and Northern blots. Statistical tests and sample sizes are given in the figure legends. Bars grouped together were not significantly different unless indicated; bars grouped separately were not compared to one another. *P* ≤ 0.05 were considered statistically significant.

## Supplementary Material

Supplementary File

Supplementary File

Supplementary File

Supplementary File

Supplementary File

Supplementary File

Supplementary File

Supplementary File

Supplementary File

Supplementary File

## Data Availability

The data reported in this paper have been deposited in the Gene Expression Omnibus (GEO) database (accession no. GSE176183).
