# Supplementary material for: The Clp1 R140H mutation alters tRNA metabolism and mRNA 3′ processing in mouse models of pontocerebellar hypoplasia
Source: Proc Natl Acad Sci U S A. 2021 Sep 21;118(39):e2110730118. doi: 10.1073/pnas.2110730118 (PMC8488643; doi:10.1073/pnas.2110730118)
Supplement: Supplementary File [file pnas.2110730118.sapp.pdf]

PNAS
www.pnas.org

Supplementary Information for

The *Clp1* R140H mutation alters tRNA metabolism and mRNA 3’ processing in mouse models of pontocerebellar hypoplasia

Caitlin E. Monaghan[a,b], Scott I. Adamson[c,d], Mridu Kapur[a,b], Jeffrey H. Chuang[c,d], and Susan L. Ackerman[a,b,1]

[a]Department of Cellular and Molecular Medicine, Division of Biological Sciences, Section of Neurobiology, University of California San Diego, La Jolla, CA 92093

[b]HHMI, University of California San Diego, La Jolla, CA 92093

[c]The Jackson Laboratory for Genomic Medicine, Farmington, CT 06030

[d]Department of Genetics and Genome Sciences, Institute for Systems Genomics, UConn Health, Farmington, CT 06030

[1]To whom correspondence may be addressed. Email: sackerman@ucsd.edu.

This PDF file includes:

Supplementary text
Figures S1 to S7
Tables S1 to S6
Legends for Datasets S1 to S9
SI References

Other supplementary materials for this manuscript include the following:

Datasets S1 to S9

Supplementary Text

Isolation of Motor Neuron RNA. First, 2.5 mg papain (Worthington Biochemical Corp.) was resuspended in 200 µl activation solution [1.1 mM EDTA, 67 µM β-mercaptoethanol, and 5.5 mM cysteine in HBSS (Gibco 14175-095)] and incubated at 37 ˚C for 30 min. Activated papain was supplemented with 500 µl warmed complete DH medium [0.18 mM adenine, 625 µM HCl, and 10% FBS in a 3:1 mix of DMEM (Life Technologies 11995-065) and Ham's F-12 (11765-054)] and 550 µl warmed HBSS, and filter sterilized with a 0.2 µM filter. Each pup was decapitated, its spinal column dissected, and its spinal cord hydraulically extruded with HBSS into a petri dish (1). HBSS was replaced with 1 ml warmed papain solution, and the spinal cord was minced into ~ 0.5 mm pieces with a razor blade. The spinal cord and papain solution were transferred to a 15 ml conical tube connected to flowing O_2/CO_2 (95%/5%) via a 0.2 µm filter in its cap, and the tube was incubated in a 37 ˚C water bath for 20 min with agitation every 5 min. Samples were put on ice, supplemented with 3 ml chilled complete DH medium, and dissociated by being pipetted up and down six times with a 10 ml glass serological pipette. Remaining tissue fragments were allowed to settle for 1–5 min, the supernatant was transferred to a second 15 ml tube, and the dissociation and transfer steps were repeated two more times, each time with 2 ml fresh medium. The sample was pelleted at 300 x g for 10 min at 4 ˚C and resuspended in 1 ml FACS buffer [100 U/ml rRNasin (Promega) and 1% RNase-free BSA (Gemini Bio-Products) in 1x PBS]. Cells were strained through a 40 µm cell strainer (BD Falcon) into a 1.7 ml tube, pelleted at 300 x g for 10 min at 4 ˚C, resuspended in 1 ml FACS buffer, repelleted, resuspended in 4% PFA in 1x PBS supplemented with 100 U/ml rRNasin, and incubated on ice for 15 min. Cells were pelleted at 1,000 x g for 5 min at 4 ˚C, rinsed with 1 ml FACS buffer, resuspended in 0.3 ml FACS buffer, and frozen at –80 ˚C until cell sorting.

GFP^+ cells were collected into 1.7 ml tubes containing 0.5 ml FACS buffer with 200 U/ml rRNasin by a BD FACSAria Fusion cell sorter using a 130 µm nozzle. Cells were pelleted at 5,000 x g for 10 min at 4 ˚C, resuspended in 100 µl PKD solution (without proteinase K) from the miRNeasy FFPE kit, and frozen at –80 ˚C until RNA extraction. For RNA extraction, 6.25 µl proteinase K was added to each sample, which was then incubated at 56 ˚C in a thermomixer for 1 h. Samples were centrifuged at 20,000 x g for 20 min at 4 ˚C, the supernatants from three samples of the same genotype were combined in a new tube, 30 µl each of DNase booster buffer and DNase I stock solution were added, and samples were mixed by inversion and incubated at room temperature for 15 min. The rest of the miRNeasy protocol was followed as per the manufacturer's instructions, using 960 µl RBC buffer and 3,360 µl ethanol, and eluting with 14 µl water. Samples with RINe scores > 8 were used for library production.

3'READS+ Data Processing. Unique molecular identifiers (UMIs) were extracted from reads in 3'READS+ libraries using UMI-tools (v1.0.1) (2) extract with parameters --bc-pattern = NNN and --bc-pattern2 = NNN. Adaptors were removed using cutadapt (v1.18) (3) with parameters -a NNNNTGGAATTCTCGGGTGCCAAGG -A NNNNGATCGTCGGACTGTAGAACTCTGAAC. Poly(A) tails were then trimmed from reads using cutadapt with parameters a "A{100}"X --overlap 1 -m 20 -G X"T{100}" --overlap 2 -m 20. Reads were then mapped to mm10 using hisat2 (v2.1.0) (4) with a Gencode vM24 annotation using default parameters. Duplicated reads were collapsed using UMI-tools dedup.

In order to identify polyadenylation clusters (PACs), the following steps were taken (5). First, reads 2 of processed reads were extracted using samtools view (v1.7) (6) with parameters -f 0x80 then converted to bed files using bedtools (v2.29.2) (7) bedtobam with the -split parameter set (in order to extract splice-aware segments). In order to identify reads with untemplated A's at the 3' end, we first extended read 2 coordinates 100 nt downstream using bedtools slop (with -r 100), and extracted genomic sequences using bedtools getfasta (with -s set). FASTA entries that corresponded to spliced reads were combined using a custom Python script. Trimmed reads were then compared with untrimmed reads and the genomic reference to identify reads that had two or more untemplated A's at the 3' end using a custom Python script. These reads were then clustered into PACs using bedtools cluster with the parameters -d 25 and -s. Clusters were then filtered to remove ones with fewer than five reads when all samples were combined.

RNA-Seq Data Processing. Adaptors were trimmed with cutadapt using the following parameters: -u 1 -U 1 --minimum-length 50 -a AGATCGGAAGAG -A AGATCGGAAGAG. Trimmed read pairs were then mapped to the

mm10 genome with a Gencode vM24 annotation using hisat2 (with parameters --trim5 1 --rna-strandness RF --dta).

In preparation for annotation of novel PASs, StringTie (v2.1.4) (8) was run with default parameters on all RNA-seq data using Gencode vM24 as a reference. The resulting sample-specific annotations were merged with StringTie merge, and the resulting file was the customized transcriptome used for analysis where specified.

Alternative polyadenylation (APA) analysis was performed using LABRAT (v0.1.0) (9) with a modified annotation to reflect the PACs identified in the 3'READS+ analysis or inferred from the StringTie transcriptome assembly. To produce this modified annotation, PACs identified from the 3'READS+ data were intersected with transcripts in the StringTie reference using bedtools intersect. A custom Python script then parsed these intersections and created a new gff file containing new transcripts that terminated at each PAC intersection, so that each PAC would be considered as a potential PAS by LABRAT. To identify genes with differential APA between genotypes, LABRAT was then run on the FASTQ files, using the --lasttwoexons parameter in the makeTFfasta stage, and default parameters for runSalmon and calculatepsi steps.

To identify changes in usage of specific PASs (as opposed to LABRAT-identified APA at the gene level), we first extracted the expression of the last two exons of each transcript from the salmon (10) files produced as part of the LABRAT pipeline. Transcripts per million (TPM) estimates for each transcript end were averaged if the transcript ends were within 25 nt and had been merged by LABRAT (parsed from "numberofposfactors.txt" produced in the LABRAT pipeline). The fraction of expression for a specific PAS m in a gene with n PASs was calculated as $\boldsymbol{TPM_m / \sum_{i=1}^{n} TPM_i}$. Two generalized linear models were then used to predict the fraction of expression for a specific PAS for each sample, one with a categorical predictor of genotype, and a null model (of just a constant). A likelihood ratio test was then used to identify the significance of the goodness of fit for the genotype model compared to the null model.

To assign PASs to different features, we used bedtools intersect with Gencode vM24 annotation filtered for specific features. PASs that overlapped coding sequences or 3' UTR features were considered as such for protein coding transcripts. PASs that overlapped exon features in noncoding transcripts were considered as noncoding. If a PAS overlapped the transcript bounds, but no exon features, it was considered intronic. PASs that did not intersect Gencode annotated transcripts were considered downstream and assigned to the closest upstream reference gene that was part of the same StringTie gene.

To identify sites of intronic polyadenylation, we used an approach conceptually similar to one previously published (11). Briefly, we prepared a reference based on the PACs identified in the 3'READS+ data and considered intronic polyadenylation sites as PACs that did not overlap with exon or UTR features in Gencode vM24, but were located within gene boundaries (using bedtools intersect). We then identified the nearest upstream exon to each intronic PAC using bedtools closest with parameters (-s -fu -D a -io -t all). For PACs with an exon within 5 kb upstream, the region between the intronic PAC and the nearest upstream exon was used as a new "exon" feature and a new annotation was created using the DEXSeq (v1.32.0) (12) script dexseq_prepare_annotation.py. Read counts associated with each feature were quantified using dexseq_count.py (also associated with DEXSeq). Differential usage of features in genes with intronic PACs was identified using DEXSeq, and results were filtered to only include "exon" segments that were derived from intronic polyadenylation events.

For analyses involving neighboring genes, these neighbors were limited to genes identified by 3'READS+, although the genes of interest were not similarly restricted. Nearest genes with a specific orientation relative to a given gene were identified using bedtools closest with the following respective parameters (downstream antisense: -S -k 2 -d -iu -D a -a, downstream sense: -s -k 2 -d -iu -D a -a, upstream antisense: -S -k 2 -d -id -D a -a, upstream sense: -s -k 2 -d -id -D a -a) and filtering out self intersections. The number of neighbors within a fixed window was calculated using bedtools window with -c. For analysis of the average distance to the 10 nearest neighbors, these neighbors were identified with bedtools closest -k 11 -d -a, excluding self distances. Gene length was extracted from a Gencode vM24 gff file using awk and custom Python scripts.

Gene ontology analysis was performed with ShinyGO v0.66 (13). Genes for which differential localization correlated with ALE selection were taken from Table S2 of Taliaferro et al. (14). To identify the overlap between

these genes and those with differential ALE usage in *Clp1*$^{R140H/–}$ spinal cord, we selected genes for which LABRAT *P*-adjusted ≤ 0.05 and gene type was either “ALE” or “mixed.”

For microRNA binding site prediction in distal-shifted genes, the sequences of interest were identified by extracting genes with increased distal PAS usage in the *Clp1*$^{R140H/–}$ spinal cord (i.e., genes with *P*-adjusted ≤ 0.05, $\Delta\psi > 0$) from LABRAT outputs and identifying PASs within those genes that were used more in *Clp1*$^{R140H/–}$ spinal cord than wild-type spinal cord using the likelihood ratio test (Benjamini-Hochberg corrected *P* ≤ 0.05). The most distal PAS to meet these criteria was set as the end of the sequence of interest. If one or more PASs in the same gene had significantly increased usage in the wild-type spinal cord, the most proximal of these was considered as the beginning of the sequence of interest. If no PAS was favored in the wild-type spinal cord, the beginning of the sequence of interest was defined as the most distal PAS that was used that was upstream of the most proximal of the PASs favored in the mutant. The converse definition was used to define sequences of interest associated with proximal-shifted genes. For each gene, sequences from each transcript that overlapped with the sequence of interest were extracted using bedtools getfasta (7). These sequences were scanned for microRNA binding sites using miRDB, retaining microRNAs with prediction scores ≥ 80 (15, 16). Sequence of interest/microRNA pairs were filtered to only include genes for which all transcripts corresponding to the sequence of interest had at least one microRNA seed match. To assess enrichment of microRNA binding sites in the inter-PAS regions of distal- or proximal-shifted genes, we compared predicted microRNA binding sites as described above to a background gene set composed of the 3’ UTRs of all expressed genes lacking either increased distal or increased proximal polyadenylation, as appropriate. MicroRNA binding sites for background genes were downloaded from miRDB (version 6.0) and filtered to only include targets with a score ≥ 80 (15, 16). For each microRNA, Fisher’s exact test was used to determine if the proportion of distal- or proximal-shifted RNAs targeted by that microRNA was greater than the proportion of its targets in the background gene set.

For differential expression analysis, trimmed reads were pseudo-aligned to a Gencode vM24 transcriptome FASTA using kallisto (v0.44.0) (17) with parameters --bias and -b 100. sleuth (v0.30.0) (18) was used to identify differentially expressed genes by fitting full and null model fits to the genotype and using the Wald test to identify genes for which the full model was significantly better than the null model. sleuth β values are estimates of differential expression analogous to the natural log of the fold-change of the mutant relative to the wild-type.

Gene biotypes for each gene were retrieved using biomaRt (v2.42.1) (19). Genes detected in the 3’READS+ libraries were identified using bedtools intersect to identify “exon” features in genes (Gencode vM24) that intersected with 3’READS+ PACs, and these were used to identify the nearest genes.

To determine if there were significant overlaps between differentially expressed genes and genes with differential PAS usage, we used Fisher’s exact test, a function within the GeneOverlap R package (20). Differentially expressed genes were genes with an adjusted *P* ≤ 0.05 (down-regulated when $\beta < 0$, up-regulated when $\beta > 0$). Genes with differential PAS usage were identified as having LABRAT *P*-adjusted ≤ 0.05 (proximal-shifted when $\Delta\psi < 0$, distal-shifted when $\Delta\psi > 0$).

The sleuth β values of the protein-coding genes with predicted microRNA binding sites between their proximal and distal PASs (as defined above) were compared to those of other protein-coding genes using Mann-Whitney U tests.

Motif analysis was done with streme (v5.3.3) (21) using the parameter --rna. Upstream, middle and downstream regions were considered as 100–41 nucleotides upstream, 40–1 nucleotides upstream, and 1–100 nucleotides downstream of a PAS, respectively. To identify motifs associated with PASs used differentially between genotypes, APA isoforms with increased or decreased usage were identified as significant if the Benjamini-Hochberg-corrected *P* was ≤ 0.05 by the likelihood ratio test approach described above. APA isoforms that were not significant by this metric were considered unchanged, and used as a background motif set using the -n parameter. Parameters -minw 5 -maxw 8 were also set for this analysis. To identify motifs in up-regulated and down-regulated genes, the last PAS detected in a gene was identified using bedtools intersect of genes and PACs identified in the 3’READS+ data. Genes with adjusted *P* ≤ 0.05 and $\beta > 0$ were considered up-regulated, those with adjusted *P* ≤ 0.05 and $\beta < 0$ as down-regulated, and all others as background genes. The background genes were used as a negative motif set with the -n parameter, and -minw 6 -maxw 10 were also set.

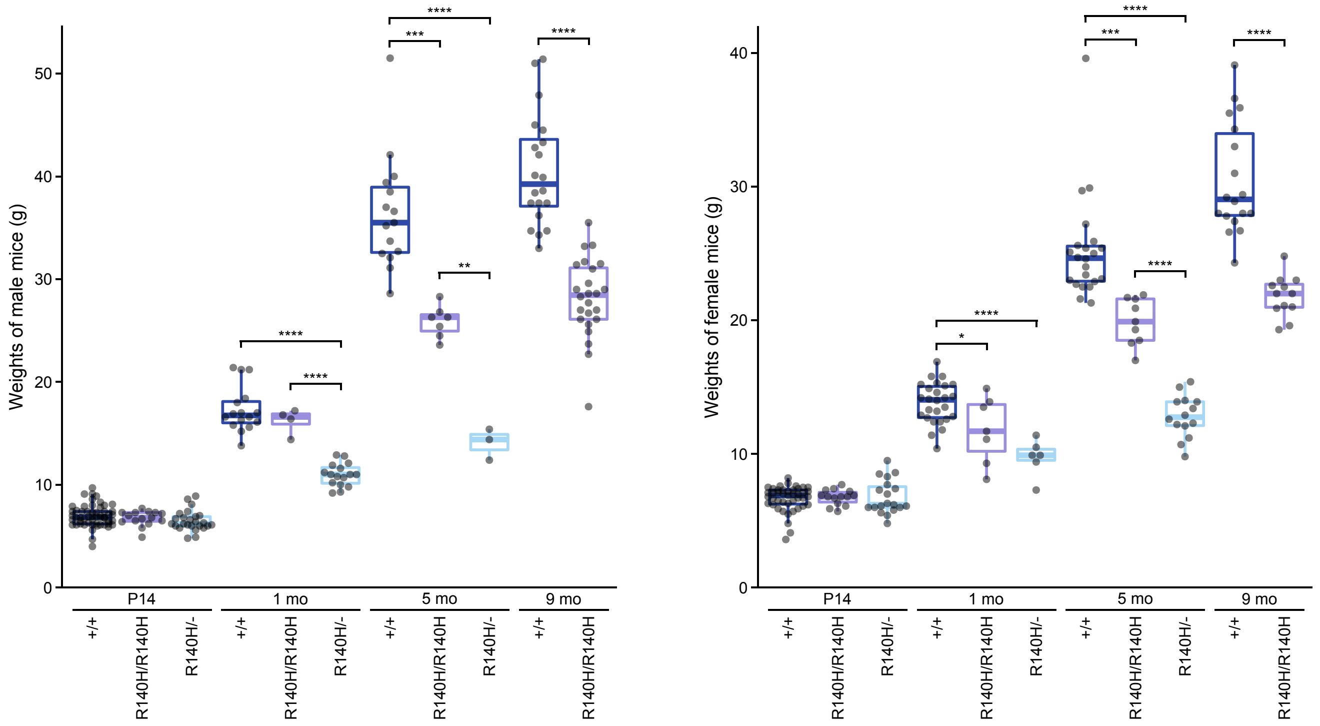

Figure S1. *Clp1* mutant mice have reduced body weights. Box plots show the median, quartiles (boxes), and range excluding outliers (whiskers). One-way ANOVA with Tukey's multiple comparisons tests (P14, 1 mo, 5 mo) or *t* tests with Welch's correction (9 mo). g, grams; *$P \leq 0.05$; **$P \leq 0.01$; ***$P \leq 0.001$; ****$P \leq 0.0001$.

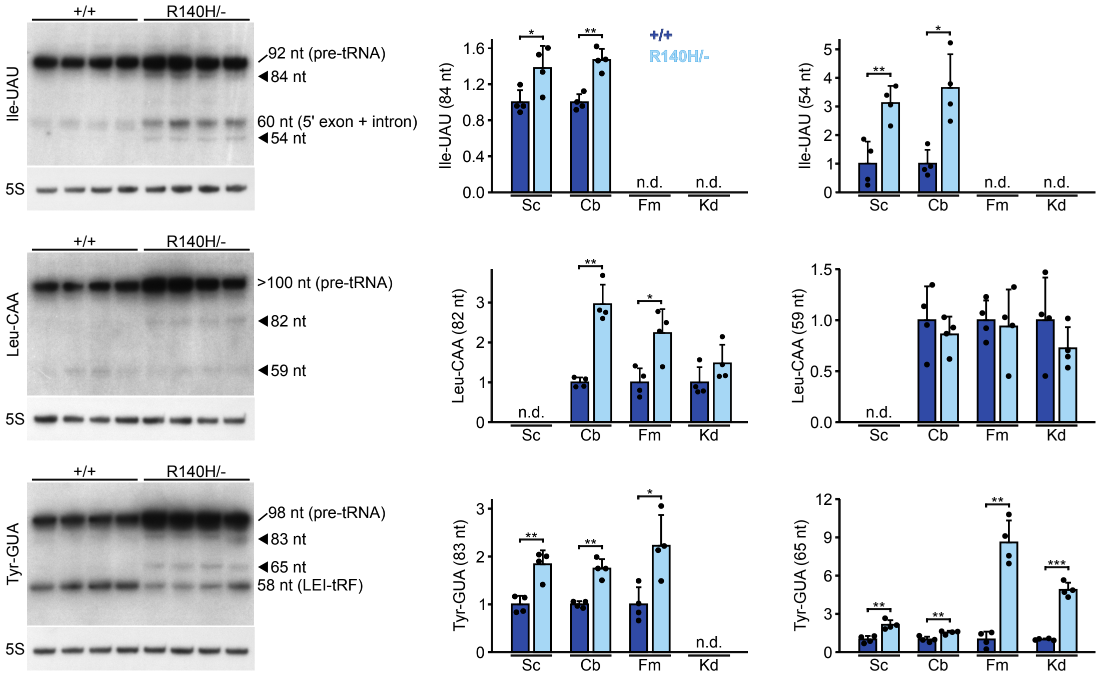

Figure S2. tRNA products recognized only by intronic probes are up-regulated in *Clp1*$^{R140H/–}$ tissues. (*Left*) Northern blots of cerebellar RNA using probes to tRNA introns. Bands not detected by 5' or 3' exon probes upon reprobing are indicated with triangles. (*Right*) Signal intensities normalized to 5S rRNA (loading control). Mean + SD. *t* tests with Welch's correction. Cb, cerebellum; Fm, forebrain/midbrain; Kd, kidney; n.d., not detected; nt, nucleotides; Sc, spinal cord; *$P \leq 0.05$; **$P \leq 0.01$; ***$P \leq 0.001$.

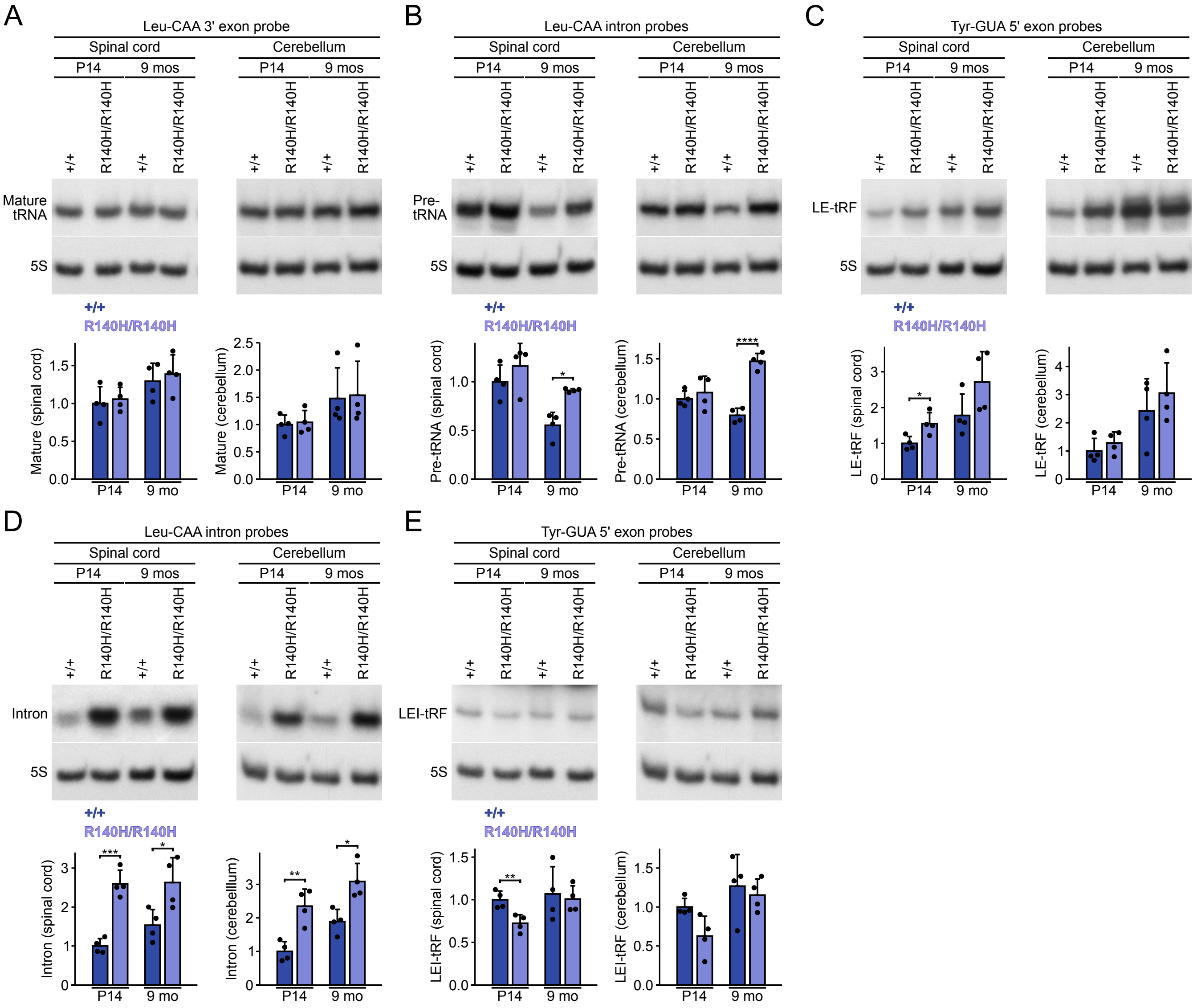

Figure S3. *Clp1*$^{R140H/R140H}$ mice recapitulate the tRNA product profile of *Clp1*$^{R140H/–}$ mice in an age-dependent manner. (*A*–*E*) Northern blots on spinal cord and cerebellar RNA showing various gene products derived from Leu-CAA and Tyr-GUA tRNA genes. Signal intensities normalized to 5S rRNA (loading control). Mean + SD. *t* tests with Welch's correction. *$P \leq 0.05$; **$P \leq 0.01$; ***$P \leq 0.001$; ****$P \leq 0.0001$.

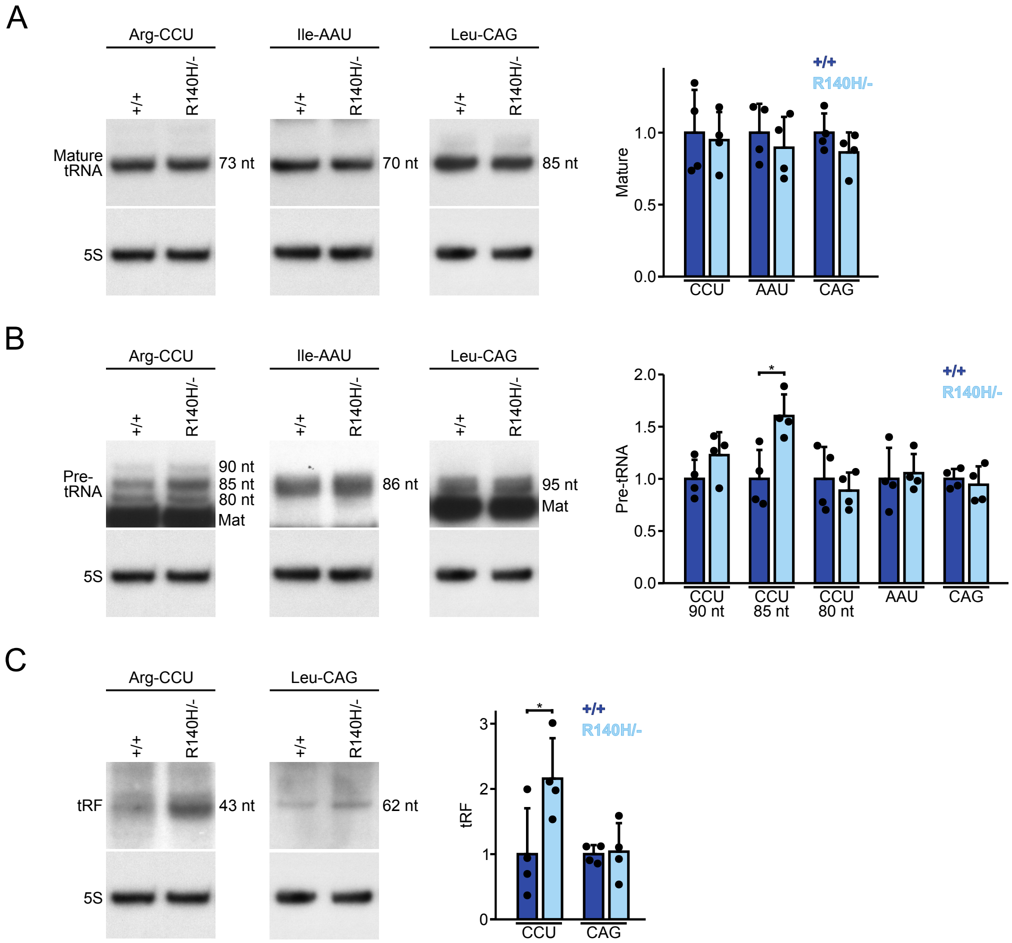

Figure S4. Levels of some pre-tRNAs and tRFs from intronless genes also vary in the *Clp1*$^{R140H/–}$ cerebellum. (*A*–*C*) Northern blots on cerebellum at P14 showing products of three families of intronless tRNA genes. Signal intensities normalized to 5S rRNA (loading control). Mean + SD. *t* tests with Welch's correction. Mat, mature tRNA; nt, nucleotides; *$P \le 0.05$.

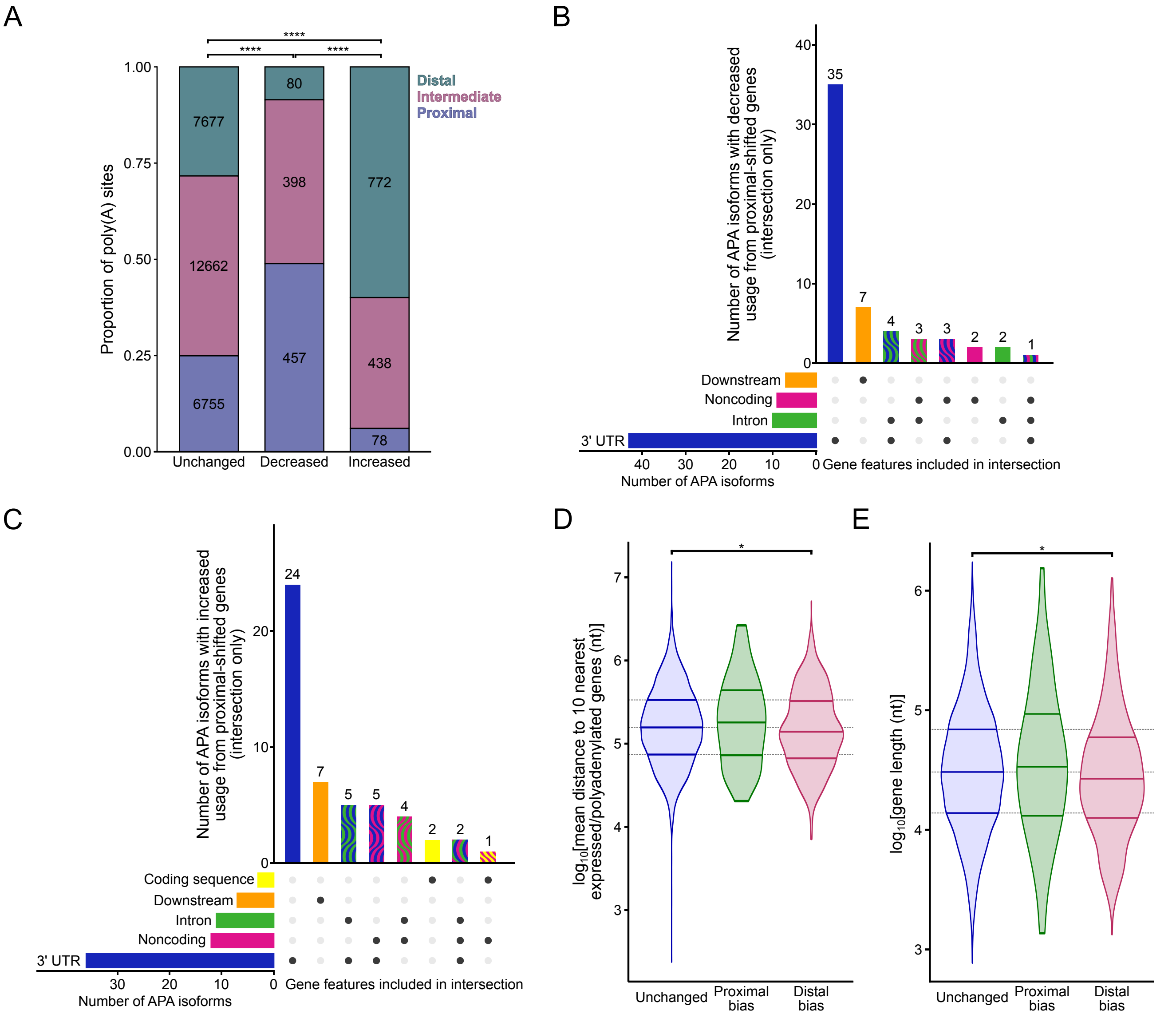

Figure S5. PAS selection is dysregulated in P14 *Clp1*$^{R140H/–}$ spinal cord. (*A*) Relative PAS positions for APA isoforms with normal or altered PAS usage in the mutant relative to the wild-type mouse. If only one APA isoform from a gene was detected by RNA-seq, it was excluded. Categorization of an APA isoform as decreased, increased, or unchanged was based on the significance and sign of the ΔPAS usage fraction (i.e., *Clp1*$^{R140H/–}$ PAS usage fraction – *Clp1*$^{+/+}$ PAS usage fraction), and differences in proportions of proximal, intermediate, or distal sites between categories were assessed using chi-squared tests. (*B* and *C*) UpSet plots showing the numbers of gene features that overlap with the PASs of differentially utilized APA isoforms (ΔPAS usage fraction ≠ 0 and *P*-adjusted ≤ 0.05) from genes with differential PAS usage (LABRAT Δψ ≠ 0 and *P*-adjusted ≤ 0.05). APA isoforms that were derived from proximal-shifted genes and show decreased usage in the mutant (*B*) and APA isoforms that were derived from proximal-shifted genes and show increased usage in the mutant (*C*). (*D* and *E*) Average distance to the ten nearest expressed and polyadenylated neighboring genes (*D*) and gene length (*E*). Unchanged indicates genes with LABRAT adjusted *P* > 0.05. Proximal bias indicates genes in which adjusted *P* ≤ 0.05 and Δψ < 0. Distal bias indicates genes in which adjusted *P* ≤ 0.05 and Δψ > 0. Mann-Whitney U tests were used to assess significance. nt, nucleotides; **P* ≤ 0.05; *****P* ≤ 0.0001.

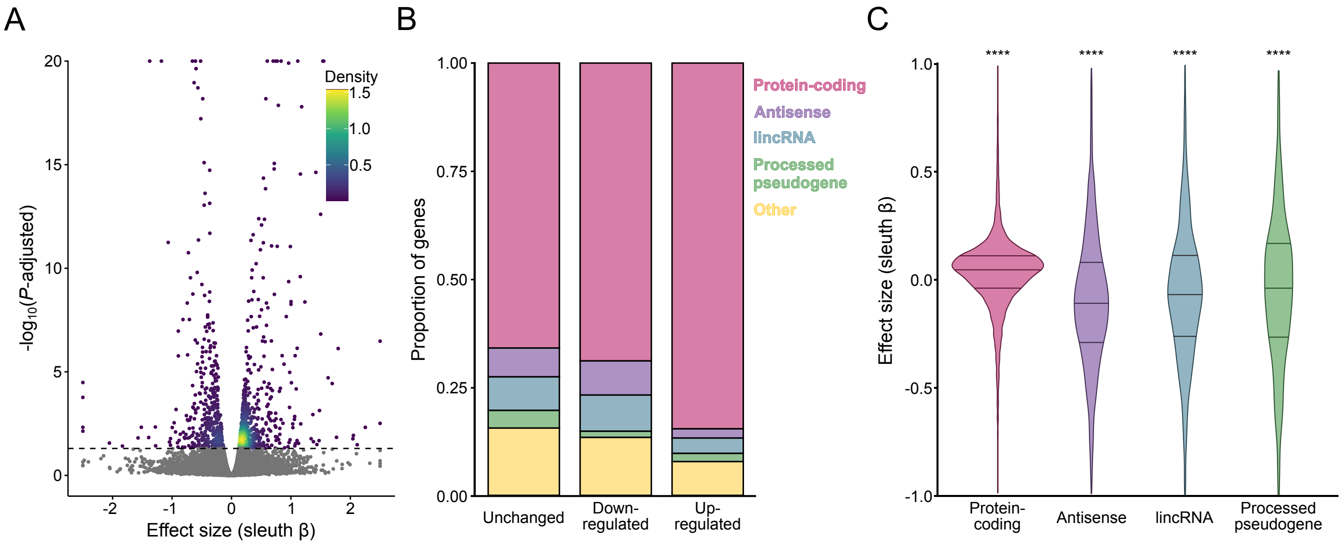

Figure S6. Gene expression is dysregulated in P14 *Clp1*$^{R140H/–}$ spinal cord. (*A*) Analysis of gene expression in mutant relative to wild-type spinal cord. sleuth β is an approximation of the natural log of the fold-change of expression in *Clp1*$^{R140H/–}$ spinal cord relative to wild-type spinal cord. Dashed line corresponds to *P*-adjusted = 0.05. Values were clipped at x = ±2.5 and y = 20. (*B*) Biotypes of genes that were down-regulated, up-regulated, or not differentially expressed. For each biotype, Fisher's exact tests were used to compare the fraction of genes that were up-regulated or down-regulated to the fraction of genes that were unchanged. For up-regulated genes, each biotype was significantly different with $P < 0.0001$ for all biotypes except processed pseudogenes (P = 0.0013). For down-regulated genes, no biotypes were significantly different except processed pseudogenes (P = 0.0060). (*C*) Effect sizes for biotypes. Mann-Whitney U tests were used to assess whether effect sizes were significantly different between each biotype and total genes. lincRNA, long intergenic noncoding RNA; nt, nucleotides, ****$P \leq 0.0001$.

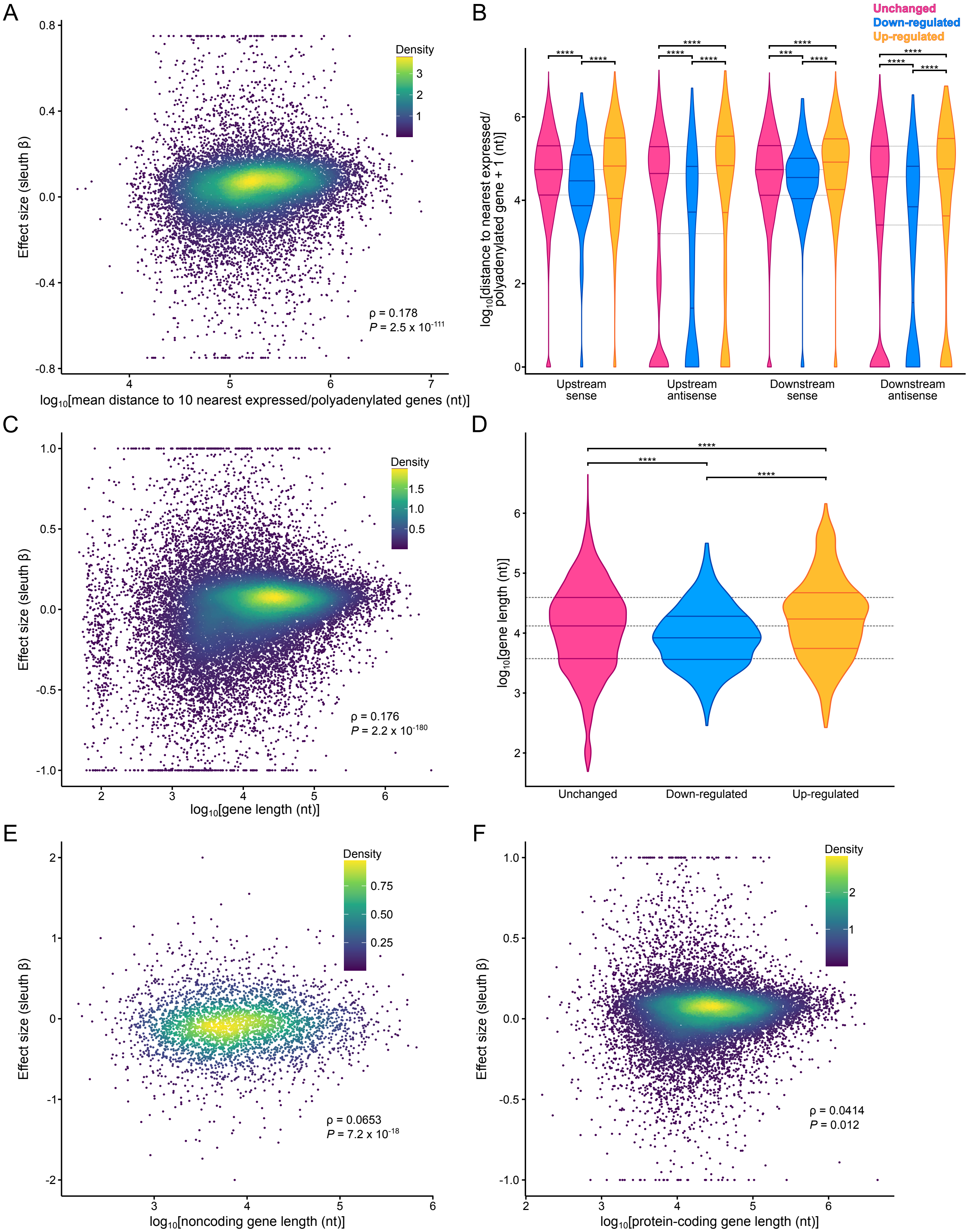
A
Density
ρ = 0.178
P = 2.5 x 10^-111
Effect size (sleuth β)
log10[mean distance to 10 nearest expressed/polyadenylated genes (nt)]
B
Unchanged
Down-regulated
Up-regulated
log10[distance to nearest expressed/polyadenylated gene + 1 (nt)]
Upstream sense
Upstream antisense
Downstream sense
Downstream antisense
C
Density
ρ = 0.176
P = 2.2 x 10^-180
Effect size (sleuth β)
log10[gene length (nt)]
D
log10[gene length (nt)]
Unchanged
Down-regulated
Up-regulated
E
Density
ρ = 0.0653
P = 7.2 x 10^-18
Effect size (sleuth β)
log10[noncoding gene length (nt)]
F
Density
ρ = 0.0414
P = 0.012
Effect size (sleuth β)
log10[protein-coding gene length (nt)]

Figure S7. Gene expression changes in the P14 *Clp1*$^{R140H/–}$ spinal cord correlate positively with intergene spacing and gene length. (*A*) Relationship of relative gene expression to gene density. Dots depict genes detected by RNA-seq (independent of detection by 3'READS+). Expressed/polyadenylated genes are defined as those identified from 3'READS+ libraries made from spinal motor neurons. Effect size values were clipped at ±0.75. (*B*) Distance of down-regulated, up-regulated, or not differentially expressed genes to the nearest genes with particular positions and orientations. All genes detected by RNA-seq were included as genes of interest, but neighboring genes were limited to expressed and polyadenylated genes (i.e., those identified by 3'READS+). Significance was assessed using Mann-Whitney U tests with Benjamini-Hochberg corrections. (*C*) Relationship of relative gene expression to gene length. Dots depict genes detected by RNA-seq. Effect size values were clipped at ±1. (*D*) Lengths of down-regulated, up-regulated, or not differentially expressed genes. Significance was assessed using Mann-Whitney U tests. (*E* and *F*) Relationships of relative gene expression to gene length for antisense RNAs, lincRNAs, and processed pseudogenes (*E*) and protein-coding genes (*F*). Effect size values were clipped at ±2 (*E*) and ±1 (*F*). Correlations were assessed by Spearman's rank correlation in (*A*, *C*, *E*, and *F*). nt, nucleotides; ***$P \leq 0.001$; ****$P \leq 0.0001$.

Table S1. Northern blot analysis of Arg-UCU tRNA products in P14 wild-type and *Clp1*$^{R140H/–}$ mice.

			Spinal cord			Cerebellum			Forebrain/midbrain			Kidney		
Band size (nt)	Possible target	Probe(s)	+/+	R140H/–	*P*-value	+/+	R140H/–	*P*-value	+/+	R140H/–	*P*-value	+/+	R140H/–	*P*-value
>>100	Leader/trailer + exons + intron = 85-87 nt + leader/trailer	3' exon	n.d.	n.d.	n.d.	1.00 ± 0.32	0.72 ± 0.53	0.41	n.d.	n.d.	n.d.	n.d.	n.d.	n.d.
>100	Leader/trailer + exons + intron = 85-87 nt + leader/trailer	3' exon	n.d.	n.d.	n.d.	1.00 ± 0.24	1.04 ± 0.23	0.82	n.d.	n.d.	n.d.	n.d.	n.d.	n.d.
100	Leader/trailer + exons + intron = 85-87 nt + leader/trailer	5' exon	1.00 ± 0.06	1.10 ± 0.08	0.090	1.00 ± 0.03	1.79 ± 0.12	0.00061	1.00 ± 0.20	1.81 ± 0.49	0.038	1.00 ± 0.16	1.18 ± 0.12	0.13
		3' exon	1.00 ± 0.07	1.10 ± 0.07	0.086	1.00 ± 0.05	1.25 ± 0.12	0.0180	n.q.	n.q.	n.q.	1.00 ± 0.15	1.33 ± 0.13	0.014
		Premature	1.00 ± 0.07	1.06 ± 0.08	0.27	1.00 ± 0.05	1.40 ± 0.11	0.0017	1.00 ± 0.11	2.05 ± 0.46	0.017	1.00 ± 0.11	1.21 ± 0.08	0.023
90	Leader/trailer + exons + intron = 85-87 nt + leader/trailer	5' exon	1.00 ± 0.12	1.11 ± 0.14	0.27	1.00 ± 0.07	1.64 ± 0.14	0.00065	1.00 ± 0.19	1.38 ± 0.42	0.17	1.00 ± 0.12	1.23 ± 0.17	0.078
		3' exon	n.q.	n.q.	n.q.	1.00 ± 0.11	1.17 ± 0.15	0.14	n.q.	n.q.	n.q.	n.q.	n.q.	n.q.
		Premature	1.00 ± 0.15	1.27 ± 0.19	0.070	1.00 ± 0.08	1.56 ± 0.12	0.00036	1.00 ± 0.12	1.94 ± 0.43	0.018	1.00 ± 0.23	1.17 ± 0.18	0.30
82		3' exon	n.q.	n.q.	n.q.	1.00 ± 0.09	1.09 ± 0.16	0.36	n.d.	n.d.	n.d.	n.d.	n.d.	n.d.
74	Mature = 76 nt	5' exon	1.00 ± 0.14	0.97 ± 0.31	0.87	1.00 ± 0.13	0.92 ± 0.11	0.40	1.00 ± 0.15	0.91 ± 0.24	0.54	1.00 ± 0.17	1.40 ± 0.13	0.0096
		3' exon	1.00 ± 0.07	1.10 ± 0.46	0.69	1.00 ± 0.08	0.93 ± 0.14	0.44	1.00 ± 0.25	1.10 ± 0.42	0.69	1.00 ± 0.14	1.13 ± 0.13	0.22
61	Intron + 3' exon + trailer = 48-50 nt + trailer	3' exon	1.00 ± 0.10	1.08 ± 0.24	0.57	1.00 ± 0.10	0.89 ± 0.09	0.14	n.q.	n.q.	n.q.	1.00 ± 0.11	1.08 ± 0.13	0.41
56	Leader + 5' exon + intron = 49-51 nt + leader	5' exon	1.00 ± 0.11	0.75 ± 0.17	0.055	1.00 ± 0.09	0.61 ± 0.20	0.024	1.00 ± 0.24	0.94 ± 0.48	0.83	1.00 ± 0.25	1.04 ± 0.20	0.79
		Premature	1.00 ± 0.06	0.71 ± 0.12	0.011	1.00 ± 0.10	0.46 ± 0.21	0.0081	n.q.	n.q.	n.q.	1.00 ± 0.36	1.05 ± 0.14	0.81
51	Intron + 3' exon = 48-50 nt or 3' exon + trailer = 37 nt + trailer	3' exon	1.00 ± 0.13	0.77 ± 0.29	0.21	n.d.	n.d.	n.d.	n.d.	n.d.	n.d.	n.d.	n.d.	n.d.
50	5' exon + intron = 49-51 nt	5' exon	n.q.	n.q.	n.q.	1.00 ± 0.24	0.60 ± 0.33	0.098	n.d.	n.d.	n.d.	n.d.	n.d.	n.d.
48	Intron + 3' exon = 48-50 nt or 3' exon + trailer = 37 nt + trailer	3' exon	n.q.	n.q.	n.q.	1.00 ± 0.07	0.80 ± 0.18	0.11	1.00 ± 0.15	0.93 ± 0.16	0.55	1.00 ± 0.06	1.65 ± 0.13	0.00087
43	Leader + 5' exon = 37 nt + leader	5' exon	1.00 ± 0.18	1.17 ± 0.24	0.31	1.00 ± 0.13	1.01 ± 0.37	0.98	1.00 ± 0.11	1.04 ± 0.17	0.69	1.00 ± 0.12	2.40 ± 0.19	4.6×10^{-5}
43	3' exon + trailer = 36 nt + trailer	3' exon	1.00 ± 0.27	0.86 ± 0.59	0.69	1.00 ± 0.08	0.93 ± 0.09	0.34	1.00 ± 0.22	1.29 ± 0.19	0.092	1.00 ± 0.30	1.17 ± 0.46	0.57
38	5' exon = 37 nt	5' exon	1.00 ± 0.32	1.39 ± 0.37	0.16	1.00 ± 0.25	1.01 ± 0.22	0.94	1.00 ± 0.16	0.52 ± 0.20	0.0092	n.d.	n.d.	n.d.

Mean ± SD. *t* tests with Welch's correction, *n* = 4. The 3' CCA is accounted for in the expected size for the mature species, but not other species. Bands that could not be quantified in any tissue are not shown. n.d., not detected; n.q., not quantified (due to insufficient levels or proximity to a darker band); nt, nucleotides.

Table S2. Northern blot analysis of Ile-UAU tRNA products in P14 wild-type and *Clp1*$^{R140H/–}$ mice.

			Spinal cord			Cerebellum			Forebrain/midbrain			Kidney		
Band size (nt)	Possible target	Probe(s)	+/+	R140H/–	*P*-value	+/+	R140H/–	*P*-value	+/+	R140H/–	*P*-value	+/+	R140H/–	*P*-value
>>100	Leader/trailer + exons + intron = 93-96 nt + leader/trailer	3' exon	1.00 ± 0.12	0.70 ± 0.22	0.064	1.00 ± 0.24	0.93 ± 0.28	0.74	n.d.	n.d.	n.d.	n.d.	n.d.	n.d.
>100	Leader/trailer + exons + intron = 93-96 nt + leader/trailer	3' exon	n.d.	n.d.	n.d.	1.00 ± 0.13	0.79 ± 0.24	0.19	n.d.	n.d.	n.d.	n.d.	n.d.	n.d.
>100	Leader/trailer + exons + intron = 93-96 nt + leader/trailer	3' exon	n.d.	n.d.	n.d.	1.00 ± 0.09	0.93 ± 0.13	0.41	n.d.	n.d.	n.d.	n.d.	n.d.	n.d.
92	Exons + intron = 93-96 nt	5' exon	1.00 ± 0.09	1.06 ± 0.14	0.53	1.00 ± 0.11	1.03 ± 0.21	0.79	1.00 ± 0.08	1.45 ± 0.19	0.012	1.00 ± 0.24	0.98 ± 0.07	0.89
		3' exon	1.00 ± 0.11	1.05 ± 0.13	0.62	1.00 ± 0.05	1.18 ± 0.14	0.085	1.00 ± 0.05	1.22 ± 0.05	0.0010	1.00 ± 0.43	1.13 ± 0.02	0.58
		Intron	1.00 ± 0.13	1.14 ± 0.16	0.22	1.00 ± 0.12	1.37 ± 0.26	0.061	1.00 ± 0.21	1.63 ± 0.30	0.017	1.00 ± 0.28	0.92 ± 0.11	0.64
84	-	Intron	1.00 ± 0.13	1.38 ± 0.25	0.045	1.00 ± 0.09	1.47 ± 0.12	0.0012	n.d.	n.d.	n.d.	n.d.	n.d.	n.d.
76	-	5' exon	1.00 ± 0.14	0.94 ± 0.12	0.54	1.00 ± 0.14	0.81 ± 0.15	0.11	n.q.	n.q.	n.q.	1.00 ± 0.14	0.89 ± 0.12	0.29
71	Mature = 77 nt	5' exon	1.00 ± 0.09	1.01 ± 0.16	0.94	1.00 ± 0.22	0.92 ± 0.11	0.56	1.00 ± 0.11	1.27 ± 0.15	0.030	n.d.	n.d.	n.d.
		3' exon	1.00 ± 0.22	0.94 ± 0.28	0.74	1.00 ± 0.28	1.01 ± 0.04	0.95	1.00 ± 0.12	1.17 ± 0.22	0.23	1.00 ± 0.23	0.97 ± 0.32	0.90
60	5' exon + intron = 57-60 nt	5' exon	n.d.	n.d.	n.d.	1.00 ± 0.26	1.39 ± 0.27	0.083	n.d.	n.d.	n.d.	n.q.	n.q.	n.q.
		Intron	1.00 ± 0.37	1.65 ± 0.36	0.046	1.00 ± 0.13	2.79 ± 0.55	0.0057	n.d.	n.d.	n.d.	1.00 ± 0.80	1.18 ± 0.11	0.68
54	-	Intron	1.00 ± 0.77	3.12 ± 0.60	0.0056	1.00 ± 0.48	3.65 ± 1.18	0.014	n.d.	n.d.	n.d.	n.d.	n.d.	n.d.
45	Leader + 5' exon = 38 nt + leader	5' exon	n.d.	n.d.	n.d.	1.00 ± 0.51	0.42 ± 0.37	0.12	n.q.	n.q.	n.q.	n.q.	n.q.	n.q.
44	3' exon + trailer = 36 nt + trailer	3' exon	1.00 ± 0.16	1.11 ± 0.27	0.52	1.00 ± 0.25	0.70 ± 0.21	0.12	1.00 ± 0.27	0.71 ± 0.18	0.14	1.00 ± 0.54	0.70 ± 0.34	0.39
43	Leader + 5' exon = 38 nt + leader	5' exon	1.00 ± 0.56	1.14 ± 0.16	0.65	1.00 ± 0.27	0.58 ± 0.26	0.066	1.00 ± 0.13	0.65 ± 0.30	0.099	1.00 ± 0.23	1.38 ± 0.23	0.056
42	3' exon + trailer = 36 nt + trailer	3' exon	n.d.	n.d.	n.d.	1.00 ± 0.14	0.94 ± 0.11	0.54	n.d.	n.d.	n.d.	n.d.	n.d.	n.d.
19	Intron = 19-22 nt	Intron	1.00 ± 0.27	5.30 ± 1.58	0.011	1.00 ± 0.20	3.65 ± 0.58	0.0014	1.00 ± 0.24	3.85 ± 0.92	0.0064	1.00 ± 0.49	6.30 ± 1.56	0.0043
15	Circularized intron = 19-22 nt	Intron	1.00 ± 0.19	3.04 ± 1.30	0.050	1.00 ± 0.26	2.51 ± 0.71	0.018	n.d.	n.d.	n.d.	n.d.	n.d.	n.d.

Mean ± SD. *t* tests with Welch's correction, *n* = 4. The 3' CCA is accounted for in the expected size for the mature species, but not other species. Bands that could not be quantified in any tissue are not shown. n.d., not detected; n.q., not quantified (due to insufficient levels or proximity to a darker band); nt, nucleotides.

Table S3. Northern blot analysis of Leu-CAA tRNA products in P14 wild-type and *Clp1*$^{R140H/–}$ mice.

			Spinal cord			Cerebellum			Forebrain/midbrain			Kidney		
Band size (nt)	Possible target	Probe(s)	+/+	R140H/–	*P*-value	+/+	R140H/–	*P*-value	+/+	R140H/–	*P*-value	+/+	R140H/–	*P*-value
>> 100	Leader/trailer + exons + intron = 105-107 nt + leader/trailer	3' exon	1.00 ± 0.12	1.04 ± 0.17	0.73	1.00 ± 0.10	1.21 ± 0.17	0.089	1.00 ± 0.22	0.87 ± 0.10	0.36	1.00 ± 0.04	0.97 ± 0.19	0.79
> 100	Exons + intron = 105-107 nt	5' exon	1.00 ± 0.17	1.38 ± 0.20	0.032	1.00 ± 0.15	1.93 ± 0.30	0.0040	1.00 ± 0.08	1.33 ± 0.20	0.041	1.00 ± 0.16	0.81 ± 0.18	0.16
		3' exon	1.00 ± 0.14	1.04 ± 0.13	0.66	1.00 ± 0.14	1.73 ± 0.09	0.00026	n.q.	n.q.	n.q.	1.00 ± 0.16	0.79 ± 0.14	0.11
		Intron	1.00 ± 0.14	1.38 ± 0.16	0.013	1.00 ± 0.22	2.27 ± 0.32	0.00097	1.00 ± 0.17	1.53 ± 0.20	0.0070	1.00 ± 0.30	0.83 ± 0.26	0.42
>100	-	5' exon	n.q.	n.q.	n.q.	1.00 ± 0.15	0.93 ± 0.01	0.42	n.d.	n.d.	n.d.	n.q.	n.q.	n.q.
87	Mature = 86-87 nt	5' exon	1.00 ± 0.09	1.17 ± 0.15	0.10	1.00 ± 0.25	0.95 ± 0.15	0.73	1.00 ± 0.21	0.83 ± 0.13	0.23	1.00 ± 0.20	1.03 ± 0.22	0.85
		3' exon	1.00 ± 0.12	1.26 ± 0.13	0.028	1.00 ± 0.20	0.82 ± 0.14	0.21	1.00 ± 0.21	0.86 ± 0.08	0.27	1.00 ± 0.24	0.76 ± 0.15	0.15
82	-	Intron	n.d.	n.d.	n.d.	1.00 ± 0.12	2.96 ± 0.49	0.0028	1.00 ± 0.35	2.24 ± 0.60	0.017	1.00 ± 0.38	1.48 ± 0.47	0.17
62	5' exon + intron = 59 - 61 nt	5' exon	n.q.	n.q.	n.q.	1.00 ± 0.34	1.13 ± 0.32	0.61	n.d.	n.d.	n.d.	n.d.	n.d.	n.d.
59	-	Intron	n.d.	n.d.	n.d.	1.00 ± 0.33	0.86 ± 0.17	0.49	1.00 ± 0.19	0.94 ± 0.36	0.78	1.00 ± 0.42	0.72 ± 0.21	0.30
58	-	3' exon	n.d.	n.d.	n.d.	1.00 ± 0.22	0.75 ± 0.13	0.12	n.q.	n.q.	n.q.	1.00 ± 0.11	1.00 ± 0.26	0.98
54	3' exon + trailer = 45-46 nt + trailer	3' exon	1.00 ± 0.39	1.10 ± 0.53	0.76	1.00 ± 0.21	0.71 ± 0.24	0.12	n.q.	n.q.	n.q.	1.00 ± 0.37	0.89 ± 0.25	0.65
52	3' exon + trailer = 45-46 nt + trailer	3' exon	1.00 ± 0.79	1.33 ± 0.98	0.61	n.q.	n.q.	n.q.	n.d.	n.d.	n.d.	1.00 ± 0.35	0.63 ± 0.16	0.12
50	-	5' exon	1.00 ± 0.60	0.97 ± 0.53	0.94	1.00 ± 0.32	0.74 ± 0.27	0.25	n.q.	n.q.	n.q.	1.00 ± 0.53	1.00 ± 0.42	1.00
42	Leader + 5' exon = 38 nt + leader	5' exon	1.00 ± 0.37	1.43 ± 0.39	0.15	1.00 ± 0.22	1.02 ± 0.51	0.94	1.00 ± 0.27	0.85 ± 0.28	0.48	1.00 ± 0.44	1.13 ± 0.20	0.62
36	-	3' exon	1.00 ± 0.39	1.41 ± 0.59	0.29	1.00 ± 0.36	1.25 ± 0.34	0.36	1.00 ± 0.38	0.86 ± 0.26	0.57	1.00 ± 0.69	0.47 ± 0.18	0.23
34	-	3' exon	1.00 ± 0.56	1.44 ± 0.99	0.47	1.00 ± 0.44	0.88 ± 0.40	0.71	n.d.	n.d.	n.d.	n.q.	n.q.	n.q.
33	-	3' exon	n.q.	n.q.	n.q.	1.00 ± 0.38	0.69 ± 0.17	0.21	n.d.	n.d.	n.d.	n.d.	n.d.	n.d.
20	Intron = 21-23 nt	Intron	1.00 ± 0.37	3.81 ± 0.53	0.00023	1.00 ± 0.28	3.06 ± 0.64	0.0038	1.00 ± 0.20	1.69 ± 0.20	0.0027	1.00 ± 0.29	5.78 ± 0.35	1.1×10^{-6}
16	Circularized intron = 21-23 nt	Intron	n.d.	n.d.	n.d.	1.00 ± 0.22	1.02 ± 0.64	0.95	n.d.	n.d.	n.d.	n.d.	n.d.	n.d.

Mean ± SD. *t* tests with Welch's correction, *n* = 4. The 3' CCA is accounted for in the expected size for the mature species, but not other species. Bands that could not be quantified in any tissue are not shown. n.d., not detected; n.q., not quantified (due to insufficient levels or proximity to a darker band); nt, nucleotides.

Table S4. Northern blot analysis of Tyr-GUA tRNA products in P14 wild-type and *Clp1*$^{R140H/–}$ mice.

			Spinal cord			Cerebellum			Forebrain/midbrain			Kidney		
Band size (nt)	Possible target	Probe(s)	+/+	R140H/–	*P*-value	+/+	R140H/–	*P*-value	+/+	R140H/–	*P*-value	+/+	R140H/–	*P*-value
>>100	Leader/trailer + exons + intron = 86-93 nt + leader/trailer	5' exon	1.00 ± 0.67	1.59 ± 0.66	0.26	1.00 ± 0.08	0.94 ± 0.04	0.26	n.d.	n.d.	n.d.	n.d.	n.d.	n.d.
98	Leader/trailer + exons + intron = 86-93 nt + leader/trailer	5' exon	1.00 ± 0.19	1.26 ± 0.15	0.085	1.00 ± 0.10	2.19 ± 0.28	0.0018	1.00 ± 0.17	1.94 ± 0.48	0.024	1.00 ± 0.22	0.82 ± 0.15	0.22
		3' exon	1.00 ± 0.12	0.99 ± 0.16	0.91	1.00 ± 0.10	1.88 ± 0.27	0.0039	1.00 ± 0.05	1.99 ± 0.59	0.044	1.00 ± 0.16	0.75 ± 0.13	0.055
		Premature	1.00 ± 0.20	1.32 ± 0.16	0.050	1.00 ± 0.08	2.80 ± 0.33	0.0011	1.00 ± 0.22	2.06 ± 0.42	0.0084	1.00 ± 0.20	0.93 ± 0.14	0.59
87	-	5' exon	1.00 ± 0.23	1.12 ± 0.23	0.50	1.00 ± 0.21	0.77 ± 0.18	0.14	n.d.	n.d.	n.d.	n.q.	n.q.	n.q.
		3' exon	1.00 ± 0.17	0.85 ± 0.10	0.20	1.00 ± 0.15	0.97 ± 0.11	0.76	n.q.	n.q.	n.q.	1.00 ± 0.13	0.72 ± 0.09	0.017
83	-	Premature	1.00 ± 0.18	1.84 ± 0.29	0.0045	1.00 ± 0.07	1.75 ± 0.20	0.0028	1.00 ± 0.36	2.22 ± 0.64	0.023	n.d.	n.d.	n.d.
72	Mature = 76 nt	5' exon	1.00 ± 0.28	1.58 ± 0.46	0.084	1.00 ± 0.48	0.90 ± 0.28	0.73	n.d.	n.d.	n.d.	n.d.	n.d.	n.d.
		3' exon	1.00 ± 0.28	1.15 ± 0.28	0.46	1.00 ± 0.25	0.96 ± 0.16	0.79	1.00 ± 0.28	0.96 ± 0.04	0.79	1.00 ± 0.11	0.90 ± 0.06	0.17
65	-	Premature	1.00 ± 0.27	2.14 ± 0.37	0.0030	1.00 ± 0.21	1.54 ± 0.13	0.0070	1.00 ± 0.61	8.60 ± 1.72	0.0015	1.00 ± 0.07	4.88 ± 0.56	0.00071
58	Leader + 5' exon + intron = 50-57 nt + leader	5' exon	1.00 ± 0.42	1.15 ± 0.22	0.56	1.00 ± 0.17	0.55 ± 0.17	0.011	1.00 ± 0.14	0.81 ± 0.15	0.11	1.00 ± 0.24	0.68 ± 0.12	0.070
		Premature	1.00 ± 0.37	0.66 ± 0.12	0.16	1.00 ± 0.15	0.48 ± 0.21	0.0081	1.00 ± 0.13	0.55 ± 0.15	0.0046	1.00 ± 0.34	0.86 ± 0.15	0.51
51	Intron + 3' exon = 49-56 nt or 3' exon + trailer = 36 nt + trailer	3' exon	1.00 ± 0.40	1.20 ± 0.54	0.57	1.00 ± 0.14	0.93 ± 0.27	0.68	n.d.	n.d.	n.d.	1.00 ± 0.36	1.72 ± 0.12	0.023
50	Intron + 3' exon = 49-56 nt or 3' exon + trailer = 36 nt + trailer	3' exon	n.d.	n.d.	n.d.	1.00 ± 0.35	0.97 ± 0.28	0.91	n.d.	n.d.	n.d.	n.d.	n.d.	n.d.
45	Leader + 5' exon = 37 nt + leader	5' exon	1.00 ± 0.38	1.82 ± 0.52	0.048	1.00 ± 0.05	1.20 ± 0.27	0.23	1.00 ± 0.29	1.08 ± 0.28	0.71	1.00 ± 0.46	1.58 ± 0.37	0.10
42	Leader + 5' exon = 37 nt + leader	5' exon	1.00 ± 0.35	2.40 ± 0.64	0.014	1.00 ± 0.06	1.51 ± 0.46	0.12	n.d.	n.d.	n.d.	n.d.	n.d.	n.d.
42	3' exon + trailer = 36 nt + trailer	3' exon	1.00 ± 0.35	1.09 ± 0.56	0.80	1.00 ± 0.31	2.18 ± 0.50	0.010	n.d.	n.d.	n.d.	n.d.	n.d.	n.d.
37	5' exon = 37 nt	5' exon	n.q.	n.q.	n.q.	1.00 ± 0.34	1.16 ± 0.78	0.72	n.d.	n.d.	n.d.	n.d.	n.d.	n.d.

Mean ± SD. *t* tests with Welch's correction, *n* = 4. The 3' CCA is accounted for in the expected size for the mature species, but not other species. Bands that could not be quantified in any tissue are not shown. n.d., not detected; n.q., not quantified (due to insufficient levels or proximity to a darker band); nt, nucleotides.

Table S5. Motif enrichment near PASs.

		Upstream			Middle			Downstream		
		Motif	*P*-value	Sites	Motif	*P*-value	Sites	Motif	*P*-value	Sites
Differential PAS usage	Increased in R140H/–	UCCUGCGU	0.021	56/1288	AAUAAAU	0.014	530/1288	UCUUCCGAA	0.0020	9/1288
								CACGUU	0.021	24/1288
								AAUAGAAACC	0.045	11/1288
								UCUCAGUGUC	0.045	7/1288
	Decreased in R140H/–	CUCUAG	0.012	138/935	AAUAAAY	0.027	582/935			
		GAUCUC	0.048	24/935						
Differential gene expression	Up-regulated in R140H/–	AUCAUACAAU	0.045	7/722	UAUCUGN	0.0019	25/722	GUCUUUU	0.037	174/722
		CUGACUAUU	0.045	6/722	ACUCAGAUG	0.045	5/722			
					CACCUCU	0.045	7/722			
					AUAAUA	0.049	255/722			
	Down-regulated in R140H/–	UGCCAG	0.0032	200/314	CCUUAGC	0.020	5/314	GCGCCCC	0.024	29/314
		AGAUCC	0.037	29/314	GGCUSG	0.022	38/314			
		UAACCCCC	0.039	18/314						
		CUGAGAYC	0.039	9/314						
		GAGUGGYA	0.039	6/314						

The sequence around the PAS was divided into three regions: upstream, consisting of nt –100 to –41 relative to the PAS; middle, positions –40 to –1; and downstream, +1 to +100. Two independent analyses were performed: an APA isoform-level analysis comparing differentially expressed APA isoforms to APA isoforms used comparably between genotypes, and a gene-level analysis comparing differentially expressed genes to genes that did not change in expression between genotypes. For the gene-level analysis, the sequences analyzed were based on the most distal PAS of each gene. “Sites” indicates the number of sequences containing the motif relative to the number of sequences examined. N, any base; S, C or G; Y, C or U.

Table S6. Probes for Northern blots.

Target family	Target region	Target gene(s)	Sequence	Temp. (˚C)
Arginine-UCU	5' exon	1-1, 2-1, 3-1, 5-1	TAGAAGTCCAATGCGCTATCCATTGCG	55
	3' exon	1-1	GGGACTCGAACCCGGAACCTTTGAAT	42
		2-1	GGGATTCGAACCCACAACCTTTGAAT	
		3-1	GGGACTCGAACCCACAACCTTTGAAT	
		5-1	GGGACTCGAACCCGCAACCTTTGAAT	
	Intron	1-1	CCTTTGAATgccttcagcctcTAGAAGTCC	55/42*
		2-1	CTTTGAATcgctttctcgtcacTAGAAGTC	
		3-1	CTTTGAATgcctccatttgtcTAGAAGTCC	
		5-1	CTTTGAATctctcaatcatgcTAGAAGTCC	
Isoleucine-UAU	5' exon	Pan	ACCGCGCGCTAACCGATTGCGCCAC	55
	3' exon	Pan	TCGAACTCACAACCTCGGCAT	55/42*
	Intron	1-1	cgcttacgcctagcactgaca	42
		2-1	cacccgcacatatactgctg	
		2-2	cgctctcgcgcacacactgttg	
		2-3	tgctctgcatgtactgctg	
Leucine-CAA	5' exon	Pan	CTTGAGTCTGGCGCCTTAGACCACTC	55
	3' exon	Pan	AGAAGTGGGATTCGAACCCACGCCTCC	55
	Intron (original)	1-1	ccctcagagcgaggaagccatag	42
		2-1	tccccagacagggaagctaag	
		3-1	cacccgtaggtaaggcttgtcac	
		4-1	ccctcagtagaggaagcgaacg	
	Intron (alternate)	1-1	tcagagcgaggaagccatag	42
		2-1	tccccagacagggaagctaag	
		3-1	acccgtaggtaaggcttgtcac	
		4-1	tcagtagaggaagcgaacg	
Tyrosine-GUA	5' exon (original)	Pan	CTACAGTCCTCCGCTCTACCA	55/42*
	5' exon (alternate)	1-1, 1-2, 1-3, 1-4, 1-5, 2-1	GTCCTCCGCTCTACCAACTGAGCTATCGAAGG	55
		3-1, 3-2, 4-1	GTCCTCCGCTCTACCAGCTGAGCTATCGAAGG	
		5-1	GTCCTCCGCTCTACCAACTGAACTATCGAAAG	
	3' exon	Pan	TCGAACCAGCGACCTAAGGAT	42
	Intron	1-1	TAAGGATcactattctagtaactCTACAGT	42
		1-2	TAAGGATcaccacattagtagtCTACAGTC	
		1-3	CTAAGGATatcaacacctataCTACAGTCC	
		1-4	TAAGGATttctagaacaatgacCTACAGTC	
		1-5	AAGGATttccaggtcgaatactcCTACAGT	
		2-1	TAAGGATtaccatattgtactgaCTACAGT	
		3-1	TAAGGATgtccacagccacaagcCTACAGT	
		3-2	AAGGATggctgcaacgaatgtaaCTACAGT	
		4-1	AGGATgtcttctaacggggagttagCTACA	
		5-1	TAAGGATcaccaacgttaatactCTACAGT	
Arginine-CCU	-	Pan	TGGGACTCGAACCCACAATCCCTGGCTTAG	55
Isoleucine-AAU	-	Pan	TCTAACCAACTGAGCTAACCGGCC	42
Leucine-CAG	-	Pan	ACGCCTCCAGGGGAGACTGCGACCTGAAC	55
5S	-	Pan	CATCCAAGTACTAACCAGGCCCGAC	55

Each of the four tRNA families with high-confidence intron-containing members (Arg-UCU, Ile-UAU, Leu-CAA, Tyr-GUA) was targeted with separate probes recognizing the 5' exon (uppercase), the 3' exon (uppercase), and either the intron (lowercase) alone (for longer introns) or the intron flanked by 3' and 5' exonic sequence (for shorter introns). For every target region, the probe or pool of probes was designed to recognize all high-confidence intron-containing members of the isodecoder family (http://gtrnadb.ucsc.edu/ Feb. 2, 2021). In the Tyr-GUA, Ile-UAU, and Leu-CAA families, all members have introns; in the Arg-UCU family, one of five members has no intron, and probes excluded this member. tRNA probes recognizing intronless tRNAs were designed to recognize all members of the isodecoder family. Original and alternate probe sequences are supplied in cases in which probes were modified to compensate for suboptimal ^{32}P labeling associated with a 5' deoxycytidine. Temp., temperature for hybridization and washes. * Probes were hybridized and washed at either 55 ˚C or 42 ˚C; 42 ˚C was preferable.

Dataset S1. PACs identified in P0/P1 wild-type and *Clp1*$^{R140H/–}$ motor neurons by 3'READS+.

Dataset S2. Gene-level analysis of differential PAS usage between wild-type and *Clp1*$^{R140H/–}$ spinal cord at P14.

Dataset S3. APA isoform-level analysis of differential PAS usage between wild-type and *Clp1*$^{R140H/–}$ spinal cord at P14.

Dataset S4. Gene features overlapping with PASs for which both APA isoform-level and gene-level analyses demonstrated altered usage between wild-type and *Clp1*$^{R140H/–}$ spinal cord at P14.

Dataset S5. Analysis of differential intronic PAS usage between wild-type and *Clp1*$^{R140H/–}$ spinal cord at P14.

Dataset S6. Predicted microRNA binding sites in genes with increased proximal or distal PAS usage in *Clp1*$^{R140H/–}$ spinal cord relative to the wild-type spinal cord at P14.

Dataset S7. Differential expression analysis of wild-type and *Clp1*$^{R140H/–}$ spinal cord at P14.

Dataset S8. Gene ontology analysis of genes differentially expressed between wild-type and *Clp1*$^{R140H/–}$ spinal cord at P14.

Dataset S9. sleuth β and $\Delta\psi$ for genes with significant differences in both parameters between wild-type and *Clp1*$^{R140H/–}$ spinal cord at P14.

SI References

1. M. Richner, S. B. Jager, P. Siupka, C. B. Vaegter, Hydraulic Extrusion of the Spinal Cord and Isolation of Dorsal Root Ganglia in Rodents. *J. Vis. Exp.* **119**, e55226 (2017).
2. T. Smith, A. Heger, I. Sudbery, UMI-tools: Modeling sequencing errors in Unique Molecular Identifiers to improve quantification accuracy. *Genome Res.* **27**, 491–499 (2017).
3. M. Martin, Cutadapt removes adapter sequences from high-throughput sequencing reads. *EMBnet.journal* **17**, 10–12 (2011).
4. D. Kim, J. M. Paggi, C. Park, C. Bennett, S. L. Salzberg, Graph-based genome alignment and genotyping with HISAT2 and HISAT-genotype. *Nat. Biotechnol.* **37**, 907–915 (2019).
5. D. Zheng, B. Tian, "Polyadenylation site-based analysis of transcript expression by 3'READS+" in *mRNA Processing: Methods and Protocols*, Y. Shi, Ed. (Humana Press, 2017), pp. 65–77.
6. H. Li, *et al.*, The Sequence Alignment/Map format and SAMtools. *Bioinformatics* **25**, 2078–2079 (2009).
7. A. R. Quinlan, I. M. Hall, BEDTools: A flexible suite of utilities for comparing genomic features. *Bioinformatics* **26**, 841–842 (2010).
8. M. Pertea, *et al.*, StringTie enables improved reconstruction of a transcriptome from RNA-seq reads. *Nat. Biotechnol.* **33**, 290–295 (2015).
9. R. Goering, *et al.*, LABRAT reveals association of alternative polyadenylation with transcript localization, RNA binding protein expression, transcription speed, and cancer survival. *BMC Genomics* **22** (2021).
10. R. Patro, G. Duggal, M. I. Love, R. A. Irizarry, C. Kingsford, Salmon provides fast and bias-aware quantification of transcript expression. *Nat. Methods* **14**, 417–419 (2017).
11. S. J. Dubbury, P. L. Boutz, P. A. Sharp, CDK12 regulates DNA repair genes by suppressing intronic polyadenylation. *Nature* **564**, 141–145 (2018).
12. S. Anders, A. Reyes, W. Huber, Detecting differential usage of exons from RNA-seq data. *Genome Res.* **22**, 2008–2017 (2012).
13. S. X. Ge, D. Jung, D. Jung, R. Yao, ShinyGO: A graphical gene-set enrichment tool for animals and plants. *Bioinformatics* **36**, 2628–2629 (2020).
14. J. M. Taliaferro, *et al.*, Distal Alternative Last Exons Localize mRNAs to Neural Projections. *Mol. Cell* **61**, 821–833 (2016).
15. N. Wong, X. Wang, miRDB: An online resource for microRNA target prediction and functional annotations. *Nucleic Acids Res.* **43**, D146–D152 (2015).
16. W. Liu, X. Wang, Prediction of functional microRNA targets by integrative modeling of microRNA binding and target expression data. *Genome Biol.* **20**, 1–10 (2019).
17. N. L. Bray, H. Pimentel, P. Melsted, L. Pachter, Near-optimal probabilistic RNA-seq quantification. *Nat. Biotechnol.* **34**, 525–527 (2016).
18. H. Pimentel, N. L. Bray, S. Puente, P. Melsted, L. Pachter, Differential analysis of RNA-seq incorporating quantification uncertainty. *Nat. Methods* **14**, 687–690 (2017).
19. S. Durinck, *et al.*, BioMart and Bioconductor: A powerful link between biological databases and microarray data analysis. *Bioinformatics* **21**, 3439–3440 (2005).
20. L. Shen, GeneOverlap : An R package to test and visualize gene overlaps. *Sinai ISoMaM* (2021).
21. T. L. Bailey, STREME: accurate and versatile sequence motif discovery. *Bioinformatics* (2021) https:/doi.org/10.1093/bioinformatics/btab203.
